# Spatial differences in genetic diversity and northward migration suggest genetic erosion along the boreal caribou southern range limit and continued range retraction

**DOI:** 10.1002/ece3.5269

**Published:** 2019-05-26

**Authors:** Laura M. Thompson, Cornelya F. C. Klütsch, Micheline Manseau, Paul J. Wilson

**Affiliations:** ^1^ Natural Resources DNA Profiling and Forensic Centre Trent University Peterborough Ontario Canada; ^2^ Natural Resources Institute University of Manitoba Winnipeg Manitoba Canada; ^3^ Landscape Science and Technology Environment and Climate Change Canada Ottawa Ontario Canada; ^4^ Present address: U.S. Geological Survey National Climate Adaptation Science Center Reston Virginia; ^5^ Present address: Division of Environmental Research in the Barents Region Norwegian Institute of Bioeconomy Research (NIBIO) Svanvik Norway

**Keywords:** boreal caribou, boreal forest, genetic erosion, habitat fragmentation, microsatellite DNA, range retraction, *Rangifer tarandus*

## Abstract

With increasing human activities and associated landscape changes, distributions of terrestrial mammals become fragmented. These changes in distribution are often associated with reduced population sizes and loss of genetic connectivity and diversity (i.e., genetic erosion) which may further diminish a species' ability to respond to changing environmental conditions and lead to local population extinctions. We studied threatened boreal caribou (*Rangifer tarandus caribou*) populations across their distribution in Ontario/Manitoba (Canada) to assess changes in genetic diversity and connectivity in areas of high and low anthropogenic activity. Using data from >1,000 caribou and nine microsatellite loci, we assessed population genetic structure, genetic diversity, and recent migration rates using a combination of network and population genetic analyses. We used Bayesian clustering analyses to identify population genetic structure and explored spatial and temporal variation in those patterns by assembling networks based on *R*_ST_ and *F*_ST_ as historical and contemporary genetic edge distances, respectively. The Bayesian clustering analyses identified broad‐scale patterns of genetic structure and closely aligned with the *R*_ST_ network. The *F*_ST_ network revealed substantial contemporary genetic differentiation, particularly in areas presenting contemporary anthropogenic disturbances and habitat fragmentation. In general, relatively lower genetic diversity and greater genetic differentiation were detected along the southern range limit, differing from areas in the northern parts of the distribution. Moreover, estimation of migration rates suggested a northward movement of animals away from the southern range limit. The patterns of genetic erosion revealed in our study suggest ongoing range retraction of boreal caribou in central Canada.

## 1 INTRODUCTION

Anthropogenic activities are causing habitat loss and fragmentation, leading to shrinking ranges in many wildlife species (Ceballos, Ehrlich, & Dirzo, 2017; Schaefer, 2003; Schipper et al., 2008). It is estimated that more than 30% of all vertebrates have declining populations, with mammals having lost more than 30%–80% of their historical ranges globally during the last century (Ceballos et al., 2017; Schipper et al., 2008). Recent work has suggested that mammals, particularly those with large body sizes, may be more susceptible than some other taxa to habitat loss and fragmentation (Rivera‐Ortíz, Aguilar, Arizmendi, Quesada, & Oyama, 2015), highlighting the urgency for gaining a deeper comprehension of range retractions and the impact of reduced and fragmented habitat on natural populations.

Range retractions often occur when continuous populations become fragmented into increasingly isolated remnants (Laidre et al., 2018; Roques et al., 2016; Segelbacher, Höglund, & Storch, 2003; Wittmer et al., 2005), leading to reduced effective population sizes (Bouzat, 2010; Piry, Luikart, & Cornuet, 1999) and increased mating of related individuals (Neaves et al., 2015). The reduced potential for gene flow among isolated populations can also lead to higher population genetic differentiation and the loss of alleles due to genetic drift. This erosion of genetic diversity may reduce the fitness of individuals and the evolutionary potential of species and consequently increases the probability of extinction of natural populations (Bijlsma & Loeschcke, 2012; Bouzat, 2010; Mimura et al., 2017). Estimating spatial variation in genetic diversity and connectivity may provide insights on the impact of habitat fragmentation and an early detection of range retraction. However, it is important to acknowledge that signals of genetic differentiation can be the result of both historical and contemporary events. Factors, such as historical biogeographic events, may cause genetic differentiation among populations (Latch,Reding, Heffelfinger, Alcalá‐Galván, & Rhodes, 2014) and should be considered when evaluating the impact of contemporary landscape changes on the spatial patterns of genetic diversity.

**Figure 1:**
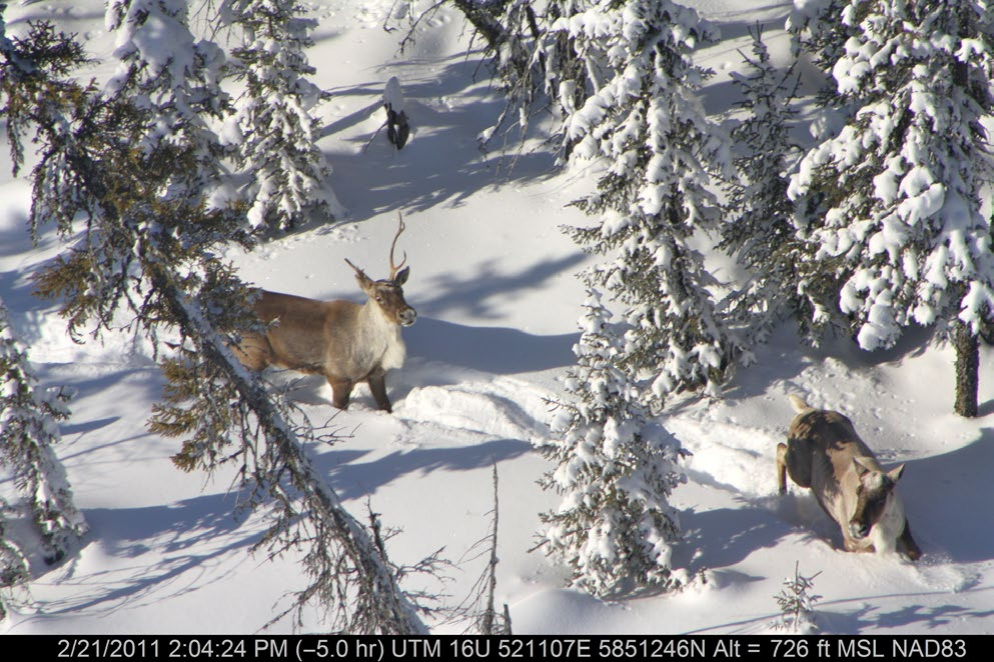
Female boreal caribou (*Rangifer tarandus caribou*) during winter in Ontario. Photograph by Gerry Racey, Ontario Ministry of Natural Resources and Forestry

Boreal woodland caribou (*Rangifer tarandus caribou;* herein referred to as boreal caribou; Figure 1) is a species with a large distribution, existing throughout the boreal zone in Canada (COSEWIC, 2011). They occur in low densities and require large areas of suitable habitat to disperse across the landscape for predator avoidance (COSEWIC, 2011). Animals move seasonally to different ranges within the boreal forest; female home ranges can vary from approximately 200 to >7,500 km^2^ (Brown, Mallory, & Rettie, 2003; Donovan, Brown, & Mallory, 2017; Racey, Harris, Gerrish, Armstrong, & McNicol, 1997; Rettie & Messier, 2001), depending on where they occur within the range (Donovan et al., 2017). These home ranges are relatively small compared with migratory populations of woodland caribou, which can have home ranges >20,000 km^2^ (Schaefer & Wilson, 2003; Wilson, 2013). Although boreal females disperse to solitary ranges during calving (Bergerud, 1988), their ranges may overlap with male/female individuals in other seasons (Darby & Pruitt, 1984; Mallory & Hillis, 1998; Rettie & Messier, 2001) and males may move >100 km during the fall rutting season (Environment Canada, 2012). Boreal caribou are at increased risk of predation because predators such as gray wolves (*Canis lupus*) have increased in density as natural and anthropogenic landscape disturbances have provided favorable conditions and increased the density of other prey species such as moose (*Alces alces*) and deer (*Odocoileus* spp.; Messier, 1994). As a result of increased human activities (e.g., forest harvesting, urban development, and oil and gas industries) in the southern part of the boreal forest, significant northward range retraction has occurred in boreal caribou (Environment Canada, 2012). Schaefer (2003) calculated that the average northward range retraction in Ontario was about 34 km per decade from 1880 to 1990, amounting to the loss of 50% of the range over that period. During the same time period, an average loss of 18 caribou wintering areas occurred per decade (Schaefer, 2003), suggesting increased local extinction rates or northward migration to core distribution ranges. More recent analyses have shown that nearly 70% of the variation in caribou recruitment is explained by natural (fire) and anthropogenic landscape disturbance (Environment Canada, 2012). Additionally, female home range sizes are more restricted in close proximity to anthropogenic activity (Wilson, Pond, Brown, & Schaefer, 2018) and declines may be continuing in certain areas, including eastern Ontario (Environment Canada, 2012) and southwestern Manitoba (Hettinga et al., 2012). Based on the decline in population sizes and distribution, the boreal Designatable Unit (DU; a species, subspecies, variety, or population(s) that is geographically or genetically discrete and evolutionarily significant; COSEWIC, 2017) of boreal caribou was federally listed as “threatened” under the Species at Risk Act in 2003 (SARA, 2003) and under provincial legislation in both Ontario (Committee on the Status of Species at Risk in Ontario; COSSARO, 2015) and Manitoba (Endangered Species and Ecosystem Act; MESEA, 2006).

Genetic erosion and isolation of populations often occur before local populations go extinct (Spielman, Brook, & Frankham, 2004). Therefore, measures of genetic diversity and connectivity may be used as an early warning signal to identify areas at risk and an early indication of potential range retraction. However, previous studies (Klütsch, Manseau, Trim, Polfus, & Wilson, 2016; Klütsch, Manseau, & Wilson, 2012) on boreal caribou have shown that some genetic variation across the region is linked to evolutionary events pre‐dating contemporary human industrial activities. Specifically, the authors found that animals in Ontario/Manitoba are descendants from three phylogenetic lineages that expanded from multiple refugia after the last glacial maximum, resulting in significant mitochondrial DNA (mtDNA) genetic differentiation across the region. Yet, no fine‐scale genetic analysis of boreal caribou in Ontario/Manitoba has been carried out to date to determine potential impacts of anthropogenic activity (while accounting for historical influences) on local population genetic structure.

The aim of this study was to assess genetic diversity and connectivity over a large and contiguous distribution of boreal caribou in central Canada in order to identify areas of genetic erosion. We used both historical and contemporary measures of genetic differentiation to distinguish naturally occurring structure (e.g., evolutionary events) from recent, human‐induced changes. We tested for genetic bottlenecks and mating among closely related individuals (i.e., inbreeding) and estimated recent migration rates. Under the hypothesis that recent anthropogenic activities increase the potential for genetic erosion, we predicted that (a) recent anthropogenic activities will cause contemporary genetic differentiation to differ from historical differentiation, (b) lower genetic diversity and connectivity and greater amounts of inbreeding and bottlenecks will be linked to populations at the southern range limit, and (c) caribou will exhibit a northward migration trend. A combination of these genetic measures is then used to identify areas at greatest risk of future population decline or extirpation.

## 2 METHODS

### 2.1 Study area and sampling

**Figure 2:**
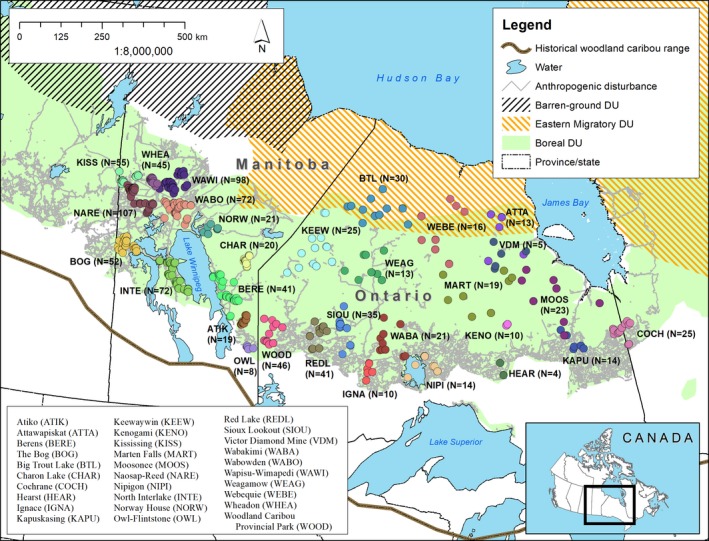
Study area showing locations of boreal caribou samples used in this study. The distribution ranges of the three recognized Designatable Units (i.e., barren‐ground, eastern migratory, and boreal caribou; COSEWIC, 2011) are shown

The study area consists of the boreal region in Ontario and Manitoba (Figure 2), including the boreal plains, boreal shield, and Hudson Plains ecozones (Wiken, 1986; for further information on ecozones and their characteristics, see Appendix S1.1).

We used the microsatellite DNA genotypes generated by Klütsch et al. (2016) sampled across the boreal caribou range in Ontario and Manitoba, Canada (1,028 unique genotypes, Figure 2). This dataset was assembled primarily from caribou fecal samples collected during winters of 2005–2011. The protocols for sample collection, DNA extraction, and genotyping can be found in Ball et al. (2007) and Klütsch et al. (2016, and references therein). Fecal samples collected during the winter generally produced relatively high yields of DNA (Ball et al., 2007), which minimized genotyping error rates (see Hettinga et al., 2012). Island populations (i.e., Slate Islands, Michipicoten Island, and Pic Island) were not included in this study as they are isolated, disjoint from the continuous mainland distribution and experiencing genetic drift (Drake et al., 2018).

### 2.2 Summary statistics for sampling locations

We examined genetic variation in the sampling locations by calculating a suite of genetic diversity statistics. Using the program GenAlEx v. 6.5 (Peakall & Smouse, 2012), number of unique alleles (*N*_a_), number of effective alleles (*N*_e_), average observed heterozygosity (*H*_o_), average expected heterozygosity (*H*_e_), sample‐size weighed expected heterozygosity (uH_e_), and inbreeding coefficient (*F*_IS_) were calculated. We used the software HP‐RARE (version 1.0, Kalinowski, 2005) to calculate allelic richness (*A*_R_) and private allelic richness (*A*_RP_; a measure of genetic distinctiveness) to correct the observed number of alleles for sample‐size differences among groups by rarefaction. Finally, we measured Euclidean distance from the center of each sampling location to the closest point along the current southern range margin to test for associations between genetic diversity values and proximity to the range margin using both Pearson's and Spearman's rank correlation coefficients. Only sampling locations with ≥10 individuals were used in the analyses.

We tested for recent reductions in effective population sizes (i.e., genetic bottlenecks) using the programs BOTTLENECK (version 1.2.02; Piry et al., 1999) and M P VAL (version 1.0; Garza & Williamson, 2001). Further information about specific run settings can be found in Appendix S1.2.

### 2.3 Bayesian clustering analyses

We used a Bayesian clustering algorithm implemented in program STRUCTURE (version 2.3.3; Pritchard, Stephens, & Donnelly, 2000) to characterize the population genetic structure. We ran the model with correlated allele frequencies and an admixture model five times with *K* (the number of populations) ranging from 1 to 10 and the burn‐in length and number of MCMC repetitions set to 500,000 and 1,000,000, respectively. We evaluated the second‐order rate of change (Δ*K*) in likelihood values (Evanno, Regnaut, & Goudet, 2005) to identify the most likely number of caribou groups and averaged the assignment values among replicates in program CLUMPP (version 1.2; Jakobsson & Rosenberg, 2007). Hierarchical structure was examined by analyzing data from Ontario and Manitoba separately using the same parameters as described for the analysis of both provinces combined; we chose these political boundaries, as opposed to identifying further substructure within existing clusters, because caribou management in this region is typically focused at the provincial level.

Chen, Durand, Forbes, and François (2007) suggested combining STRUCTURE analyses with those of a Bayesian clustering algorithm that incorporates geographic coordinates of individuals as a prior in the model. This is because spatial models may have advantages over STRUCTURE when estimating the number of populations and assigning individuals to populations, particularly when overlap among populations is low (Chen et al., 2007) or isolation by distance is present (Meirmans, 2012). Therefore, we compared STRUCTURE results with TESS results (version 2.3.1; Chen et al., 2007; Durand, Jay, Gaggiotti, & François, 2009), a spatial Bayesian clustering algorithm, to infer the number of genetic clusters (Franco̧is, Ancelet, & Guillot, 2006). First, we ran a no‐admixture model which uses a hidden Markov random field as a prior distribution with the interaction parameter set to 0.6 a total of 50 times with the maximum number of populations (*K*max) ranging from 2 to 10 and the number of MCMC iterations set to 50,000 and a burn‐in length set to 40,000. TESS calculates the deviance information criterion (DIC), a measure of both model fit and model complexity with smaller DIC values indicating a better fit of a particular model (Spiegelhalter, Best, Carlin, & Linde, 2002). For each value of *K*max, we averaged the top 5% of runs based on DIC and plotted them against *K*max. Again, the second‐order rate of change, calculated for DIC, was used to determine the most likely number of genetic groups. We then ran an admixture model (BYM) in TESS, with a linear trend a total of 50 times with the same number of iterations and burn‐in length as above to determine the proportion of each individual's ancestry that belonged to the inferred number of groups identified by the no‐admixture model (Durand et al., 2009). The assignment probabilities from the top 5% of runs (based on DIC) were averaged in CLUMPP. Again, hierarchical structure was assessed by analyzing provinces separately and combined.

### 2.4 Test for mutation effect on genetic structure

We determined whether mutations might be responsible for genetic structure in boreal caribou; an increase in the number of mutation events suggests an increased time lapse since common ancestry, which could suggest phylogeographic (or historical) structure (Hardy, Charbonnel, Fréville, & Heuertz, 2003). *R*_ST_ (Slatkin, 1995) measures genetic structure based on allele size (i.e., the number of repeats between microsatellite alleles) and is appropriate when stepwise mutations contribute to phylogeographic structure (Hardy et al., 2003). *F*_ST_ (Wright, 1965) measures genetic differentiation based on allele identity (i.e., allelic states) and is an appropriate measure when genetic drift is the primary cause of structure (Hardy et al., 2003), which can occur on recent timescales. We compared observed *R*_ST_ values between sampling locations with the distribution of *R*_ST_ obtained after 10,000 allele size permutations (*pR*_ST_; a measure analogous to *F*_ST_) using SPAGEDI (version 1.3; Hardy & Vekemans, 2002). Phylogeographic history may be an important cause of genetic differentiation when *R*_ST_ > *pR*_ST_ (i.e., *F*_ST_) across sample comparisons (Hardy et al., 2003).

### 2.5 Historical and contemporary genetic differentiation

We calculated two hierarchical analyses of molecular variance (AMOVA; Excoffier, Smouse, & Quattro, 1992) based on *R*_ST_ and *F*_ST_ to delineate historical and contemporary structure, respectively, in Arlequin (version 3.5.2.2; Excoffier & Lischer, 2010). Caribou individuals were grouped based on sampling locations, which were nested within clusters identified by the Bayesian clustering models. We assessed significance based on 10,000 permutations and allowed for 10% of missing data. Additionally, because patterns of contemporary and historical isolation by distance have the potential to generate genetic structure patterns (Dyer, Nason, & Garrick, 2010), we calculated a regression slope of both *R*_ST_ (Michalakis & Excoffier, 1996) and *F*_ST_ (Weir & Cockerham, 1984) values on spatial distances in SPAGEDI; the slope (*b*) was tested for a significant difference from zero based on 10,000 permutations, which is the equivalent of carrying out a Mantel test (Hardy & Vekemans, 2002).

### 2.6 Network analysis

We built two networks where nodes represented sampling locations and edges were weighted with either *F*_ST_ or *R*_ST_ to better understand whether sampling locations clustered based on allele size or allele state. Because the networks were constructed using genetic differentiation matrices, where every pair of nodes is connected by a unique edge, we reduced the edge set by calculating a percolation threshold (Dpe; Stauffer & Aharony, 1994) so that biologically meaningful clusters could be identified (Cowart et al., 2013; Moalic et al., 2012). Specifically, the algorithm removed edges with successively smaller edge weights and determined the point where long‐range connectivity (i.e., connectivity that spanned the size of the network) no longer existed and the network began to fragment into smaller components (Stauffer & Aharony, 1994). Using the reduced edge set, we compared the two networks based on a series of node metrics (see Appendix S1.3). Program EDENETWORKS (version 2.18; Kivelä, Arnaud‐Haond, & Saramäki, 2015) was used to construct the networks and calculate Dpe and node metrics (degree, clustering coefficient, and betweenness centrality; see Appendix S1.3) for both *F*_ST_ and *R*_ST_. Average inverse edge weight (AIEW; Koen, Bowman, & Wilson, 2016) was calculated manually.

We also used community detection algorithms to determine whether the *F*_ST_ and *R*_ST_ networks exhibited clustering patterns based on contemporary and historical processes. Community detection algorithms identify groups of nodes that are highly connected to each other and less connected to other groups of nodes in the network (Fortunato, 2010; Girvan & Newman, 2002) and have the potential to provide insights on population structure (Garroway, Bowman, Carr, & Wilson, 2008). We used eight different algorithms (see Table S1.1) and determined whether common clustering patterns emerged across techniques in both the *F*_ST_ and *R*_ST_ networks. For each community detection algorithm output, we calculated a modularity score, which can be positive or negative, with positive values indicating community structure (Newman & Girvan, 2004). Community detection algorithms and modularity scores were calculated using the igraph package (version 1.0.1; Csárdi & Nepusz, 2006) in R (R Development Core Team, 2017).

### 2.7 Recent migration rates

We used the program BIMr (version 1.0, Faubet & Gaggiotti, 2008) to estimate recent gene exchange assuming sampling occurred after reproduction and before migration among groups of individuals defined by the community detection algorithm (based on *F*_ST_) with the largest modularity score. We used 10 replicate runs with a burn‐in of 100,000 iterations, a sample size of 50,000 iterations, and a thinning interval of 100. Each run was preceded by 20 pilot runs of 1,000 iterations in an attempt to obtain acceptance rates between 25 and 45 percent. The run with the lowest Bayesian deviance (*D*_assign_) was selected to extract parameter values, including the mean, mode, and 95% highest posterior density interval (HPDI) values.

### 2.8 Mapping of genetic erosion

We used the Genetic Landscapes GIS Toolbox (Vandergast, Perry, Lugo, & Hathaway, 2011) to develop interpolated raster surfaces of genetic diversity and differentiation in ArcGIS 10.5 (ESRI). This toolbox uses the inverse distance weighting (IDW) algorithm to generate a surface from mapped genetic diversity values (Vandergast et al., 2011). To calculate a surface of genetic differentiation, the toolbox generates a network that connects the sampled areas with nonoverlapping edges. Each edge in the network has a midpoint that becomes associated with genetic differentiation values. Similar to genetic diversity, the IDW algorithm is used to generate a surface based on mapped differentiation values (Vandergast et al., 2011). We generated a genetic diversity surface based on allelic richness (*A*_R_) and a surface of contemporary genetic differentiation based on *F*_ST_ calculated among all caribou sampled areas (power = 2, variable search radius = 12 points) to identify regions exhibiting characteristics of genetic erosion.

## 3 RESULTS

### 3.1 Summary statistics for sampling locations

The mean number of alleles (*N*_a_) across sampling locations was 7.09 (ranging from 4.67 for Kenogami to 10.11 for Wapisu–Wimapedi; Table 1). Average allelic richness (*A*_R_) and private allelic richness (*A*_RP_) were lowest in North Interlake (4.21) and Kenogami (0.00), respectively, whereas both richness values were greatest for Attawapiskat (5.52 and 0.23; Table 1). The average number of effective alleles (*N*_e_) was 3.54 and was lowest for Kenogami (2.41) and greatest for Naosap–Reed (4.28; Table 1). Average observed (*H*_o_), expected (*H*_e_), and sample‐size weighted expected heterozygosity (uH_e_) across all sampled areas were 0.67, 0.69, and 0.70, respectively. *H*_o_ and uH_e_ were lowest in Kenogami (0.58 and 0.60, respectively) and greatest in Attawapiskat (0.82 and 0.77, respectively), while expected heterozygosity was also lowest in Kenogami (0.57), but greatest in Cochrane (0.74; Table 1). In general, diversity values increased as distance from the southern range periphery increased (Table 1). However, only *A*_R_ and uH_e_ exhibited a statistically significant association (*α* = 0.05) based on Pearson's correlation (*r* = 0.47, *p* = 0.013 and *r* = 0.39, *p* = 0.043, respectively); *A*_R_ also exhibited a significant association based on Spearman's rank correlation (*r* = 0.43, *p* = 0.026; Table 1). Inbreeding coefficients (*F*_IS_) ranged from −0.16 for Kapuskasing to 0.15 for Wabakimi, where larger values suggest greater inbreeding; there was not a significant association between *F*_IS_ values and distance to the range margin (Table 1). These correlation results point to genetic erosion along the range margin.

**Table 1:** Summary of genetic diversity estimates. Number of samples (*N*), number of alleles (*N*_A_), allelic richness (*A*_R_), private allelic richness (*A*_RP_), expected (*H*_E_) and observed heterozygosity (*H*_O_), unbiased heterozygosity (uHe), and inbreeding coefficient (*F*_IS_)

Sampled area	*N*	*N*_A_	*A*_R_ a, b	*A*_RP_	Ne	Ho	He	uHe^a^	*F*_IS_	Distance from southern range margin (km)
IGNA	10	4.89	4.48	0.09	3.20	0.65	0.64	0.67	−0.01	39.15
ATIK	19	6.44	4.88	0.07	3.43	0.60	0.69	0.71	0.13	44.46
BOG	52	7.56	4.89	0.14	3.55	0.70	0.70	0.71	0.01	49.34
COCH	25	7.11	5.45	0.10	4.02	0.72	0.74	0.76	0.03	61.91
NIPI	14	5.22	4.36	0.02	3.08	0.58	0.67	0.69	0.13	64.59
WOOD	46	8.11	4.79	0.09	3.36	0.64	0.68	0.69	0.08	64.90
KAPU	14	6.00	4.93	0.01	3.26	0.79	0.68	0.71	−0.16	67.56
INTE	72	6.78	4.21	0.18	3.08	0.63	0.66	0.66	0.04	75.30
BERE	41	7.67	4.92	0.14	3.65	0.65	0.69	0.70	0.06	76.54
REDL	41	7.56	4.66	0.07	3.30	0.62	0.64	0.65	0.02	80.05
SIOU	35	7.78	4.90	0.05	3.34	0.71	0.67	0.68	−0.06	111.40
KENO	10	4.67	4.27	0.00	2.41	0.58	0.57	0.60	−0.01	112.60
NARE	107	9.78	5.25	0.17	4.28	0.72	0.73	0.73	0.01	129.40
WABA	21	6.00	4.59	0.03	3.38	0.59	0.69	0.71	0.15	138.10
KISS	55	7.78	5.12	0.11	4.17	0.69	0.73	0.73	0.06	165.00
CHAR	20	6.78	4.92	0.05	3.41	0.65	0.68	0.70	0.04	186.40
WHEA	45	7.67	5.07	0.11	3.93	0.69	0.71	0.71	0.02	195.60
WABO	72	9.44	5.30	0.11	3.99	0.67	0.73	0.73	0.09	203.60
MOOS	23	6.67	4.88	0.11	3.41	0.66	0.69	0.70	0.05	204.30
MART	19	6.67	5.05	0.01	3.42	0.66	0.69	0.71	0.04	236.80
WAWI	98	10.11	5.25	0.18	3.95	0.69	0.72	0.73	0.05	241.80
NORW	21	6.89	5.01	0.09	3.60	0.68	0.69	0.70	0.02	246.20
KEEW	25	6.22	4.59	0.04	3.43	0.65	0.69	0.70	0.06	313.40
WEAG	13	5.67	4.73	0.03	3.21	0.70	0.67	0.70	−0.04	324.80
ATTA	13	6.56	5.52	0.23	4.12	0.82	0.74	0.77	−0.12	377.70
WEBE	16	7.00	5.40	0.05	3.97	0.72	0.73	0.75	0.01	413.40
BTL	30	8.44	5.24	0.12	3.66	0.68	0.70	0.71	0.04	493.60

a Significant linear association between the genetic diversity values and distance to the southern range margin based on Pearson's correlation coefficient.

b Significant monotonic association between the genetic diversity values and distance to the southern range margin based on Spearman's rank correlation coefficient.

None of the BOTTLENECK tests detected a past reduction in population size based on the stepwise mutation model (SMM) or two‐phase mutation model (TPM) (see Table S1.2). The average *M*‐ratio value (number of alleles in relation to range in allele size; Garza & Williamson, 2001) across sampled areas was 1.18 and ranged from 0.73 for the Kenogami sampled area to 1.62 for the Red Lake sampled area. The *M*‐ratio tests suggested that bottlenecks occurred only in the Kenogami sampled area and only for theta values of 0.03 and 0.3 (see Table S1.2).

### 3.2 Bayesian clustering analyses

**Figure 3:**
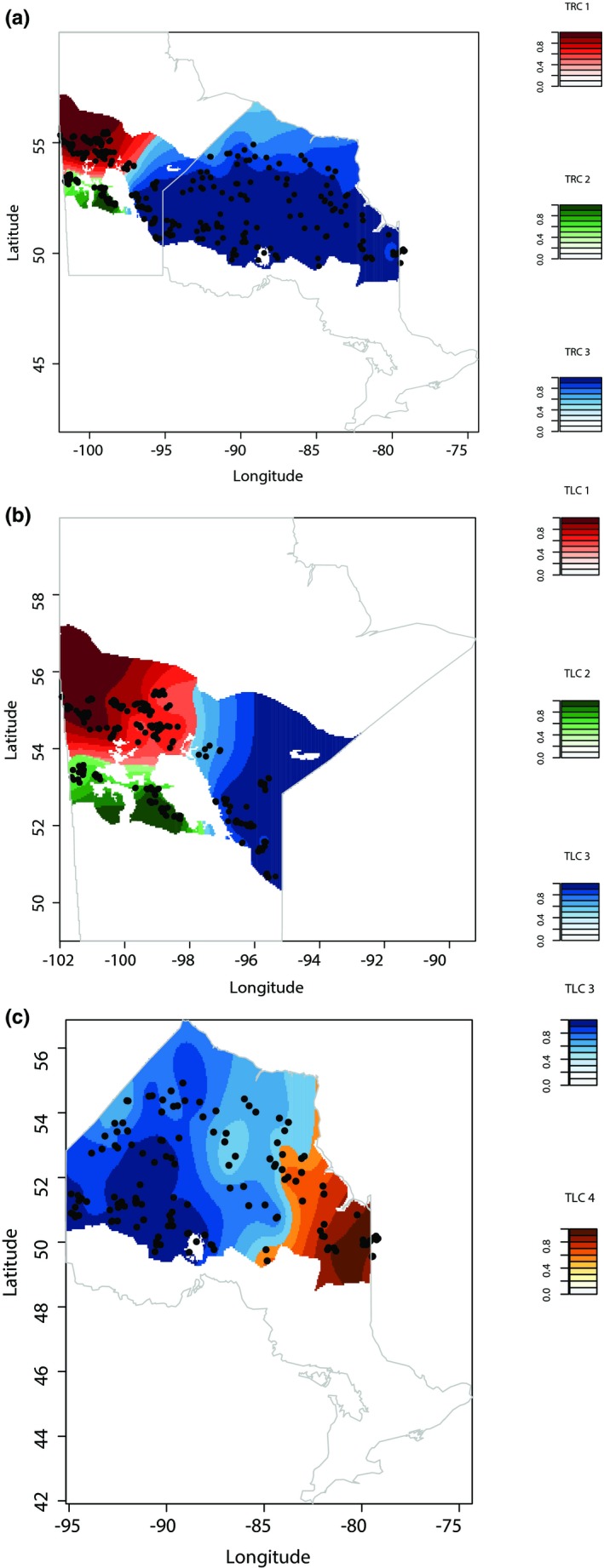
Location of woodland caribou groups inferred by the admixture model in TESS for (a) both provinces combined, (b) Manitoba, and (c) Ontario. Colors correspond to each of the inferred clusters, and darker shading indicates a greater proportion of assignment to a particular cluster. Both provinces combined are coded as TESS regional clusters (TRC), and clusters in each province are coded as TESS local clusters (TLC)

After running STRUCTURE at two scales, the results confirmed the previously identified three genetic clusters in boreal caribou (Klütsch et al., 2016; see Figures S1.1–S1.2) corresponding to a southwestern Manitoba, a northern Manitoba, and a southeastern Manitoba and Ontario cluster. The TESS analysis confirmed the same three genetic clusters when both Manitoba and Ontario populations were combined (Figure 3a, Figure S1.3). TESS did not detect additional structure when including Manitoba populations only (Figure 3b, Figure S1.3), but it did identify an additional cluster within Ontario; the additional structure separated eastern Ontario (Cochrane, Kapuskasing; TESS local cluster 4; Figure 3c, Figure S1.3) from the rest of Ontario. For a more detailed description of the clustering results, see Appendix S1.4.

### 3.3 Test for mutation effect on genetic structure

The global (observed) *R*_ST_ value of 0.041 was not significantly greater than the permuted value (*pR*_ST_) of 0.031 (*p* = 0.185), suggesting mutations did not contribute significantly to genetic differentiation at the sampling‐site level and none of the 351 pairwise permutation tests were significant after a sequential Bonferroni adjustment (Rice, 1989). When individuals were grouped based on the broader‐scale TESS regional clusters (Table S1.3), the global *R*_ST_ value of 0.056 was significantly greater than the permuted value (*pR*ST = 0.022, *p* = 0.032), suggesting that mutations partially explained genetic differentiation among genetic clusters. Additionally, 2 of the 3 pairwise comparisons were significant after sequential Bonferroni adjustment (TESS regional clusters 1 and 3, *R*_ST_ = 0.030, *pR*_ST_ = 0.009, *p* = 0.024; TESS regional clusters 2 and 3, *R*_ST_ = 0.157, *pR*_ST_ = 0.044, *p* = 0.007). When individuals were grouped based on the four TESS local clusters (Table S1.3), the global (observed) *R*_ST_ value of 0.052 was significantly greater than the *pR*_ST_ (0.022, *p* = 0.033) and a significant difference occurred between TESS local clusters 1 and 3, with an observed *R*_ST_ of 0.028 and *pR*_ST_ of 0.007 (*p* = 0.008), when considering pairwise comparisons. The results suggested that mutations indeed contributed to genetic differentiation in the study area, meaning that historical processes partially shaped contemporary genetic differentiation.

### 3.4 Historical and contemporary genetic differentiation

#### 3.4.1 *R*_ST_

The *R*_ST_ values ranged from 0.000 for 48 comparisons of sampling locations to 0.117 between Berens and The Bog (Table S1.3). The AMOVA results revealed that most of the *R*_ST_ variance was within sampling locations for both the TESS regional clusters and TESS local clusters (92.2% and 92.6%, respectively; Table 2). At other levels of variation, there was greater *R*_ST_ variance among TESS regional clusters (7.3%, *p* < 0.001; Table 2) than among sampling locations within clusters (0.5%, *p* = 0.004; Table 2). Similarly, the AMOVA calculated for the TESS local clusters showed greater *R*_ST_ variance among clusters (7.0%, *p* < 0.001; Table 2) than among sampling locations within clusters (0.5%, *p* = 0.001; Table 2). These findings suggested historical structure was aligned with the Bayesian clustering results.

**Table 2:** Hierarchical AMOVA analysis for local and regional TESS clusters (Figure 3) based on two genetic differentiation measures (i.e., *R*_ST_ and *F*_ST_)

Groupings of populations	Source of variation	*df*	*R*_ST_		*F*_ST_	
			Variance component	*p*	Variance component	*p*
TESS regional clusters	Among clusters	2	7.28	<0.001	1.62	<0.001
	Among sampling location within clusters	24	0.52	0.004	2.32	<0.001
	Within sampling locations	1887	92.19	<0.001	96.06	<0.001
TESS local clusters	Among clusters	3	6.95	<0.000	1.61	<0.001
	Among sampling locations within clusters	23	0.49	0.001	2.27	<0.001
	Within sampling locations	1887	92.55	<0.001	96.12	<0.001

Mantel tests indicated that no correlations existed between *R*_ST_ and geographic distance for the sampling locations (*b* < 0.001, *p* = 0.077), the three TESS regional clusters (*b* = 0.003, *p* = 0.829), or the four TESS local clusters (*b* = −0.002, *p* = 0.966), suggesting isolation by distance was not an important factor contributing to historical genetic structure.

#### 3.4.2 *F*_ST_

*F*_ST_ ranged from 0.000 for six comparisons of sampling locations to 0.095 between Norway House and North Interlake (Table S1.3). The AMOVA results based on *F*_ST_ suggested there was not a difference in variances between groupings based on the TESS regional clusters and the TESS local clusters (Table 2). Similar to *R*_ST_, most of the *F*_ST_ variation was in the sampled areas (96.1%, Table 2). However, the *F*_ST_ variation differed from *R*_ST_ at the other levels; variance was greater among sampling locations within clusters (2.3%, *p* = <0.001) than among clusters (1.6%, *p* < 0.001), suggesting contemporary differentiation was less aligned with the clusters than differentiation based on *R*_ST_. Similar to *R*_ST_, no correlation was detected between *F*_ST_ and geographic distance for the sampling locations (*b* < 0.001, *p* = 0.078), the three TESS regional clusters (*b* = −0.002, *p* = 0.168), or the four TESS local clusters (*b* = <0.003, *p* = 0.624), indicating that isolation by distance did not explain the genetic differentiation patterns observed.

### 3.5 Network analysis

#### 3.5.1 *R*_ST_

**Figure 4:**
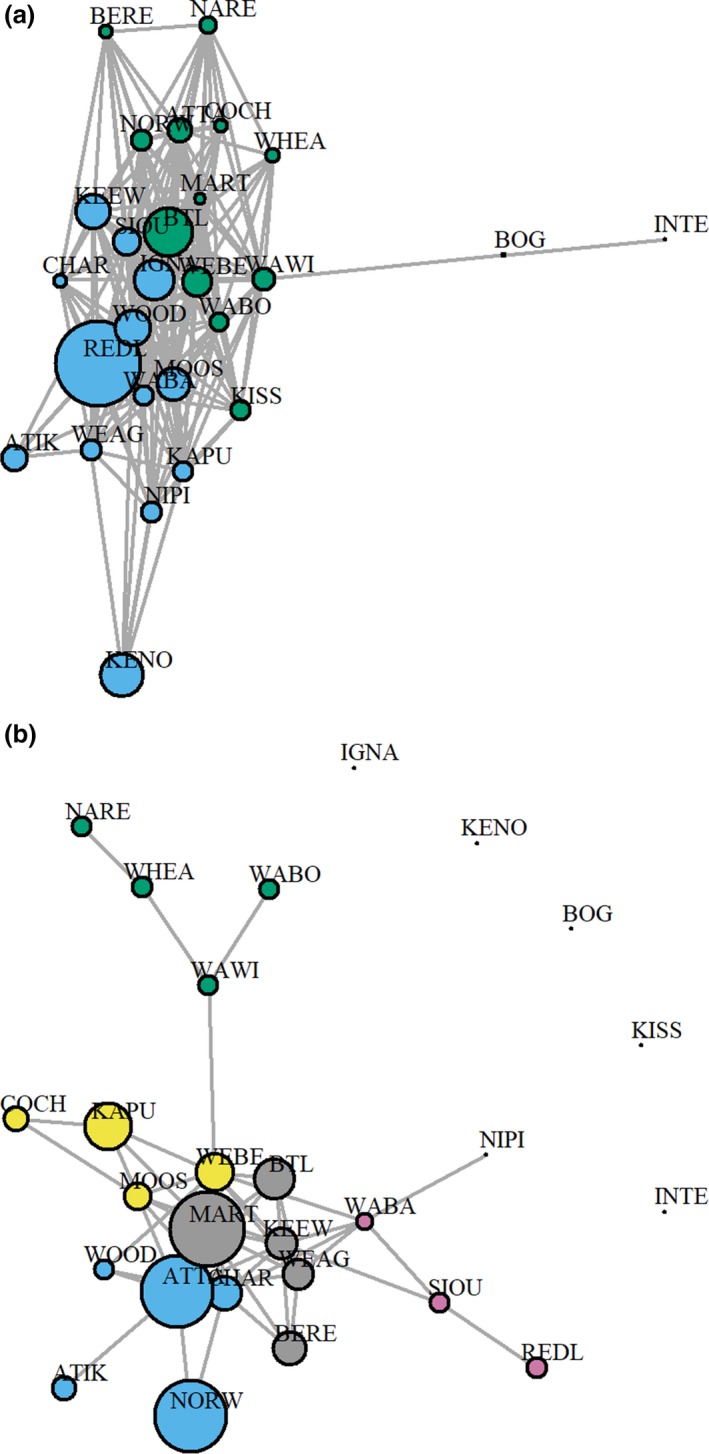
The networks constructed for (a) *R*_ST_ (leading eigenvector communities) and (b) *F*_ST_ (multilevel communities shown). The node colors represent ancestry to each community, the layout is based on Fruchterman and Reingold (1991), and node sizes represent average inverse edge weight (Koen et al., 2016). The node labels represent abbreviated sampled areas (see Figure 2)

A network weighted with *R*_ST_ was thinned based on a Dpe of 0.029 and revealed one large connected component with a large number of edges connecting Ontario and Manitoba sampling locations (Figure 4a; Table S1.4). The clusters identified by the eight different community detection algorithms exhibited some variability, but common patterns could be identified across techniques (Table S1.5). Specifically, 5 of the 8 algorithms detected a community composed of primarily Manitoba herds (some Ontario herds were also clustered with that group) and an Ontario community, including the leading eigenvector, multilevel, fastgreedy, walktrap, and spinglass community detection algorithms (Table S1.5). Additionally, the multilevel and spinglass algorithms identified The Bog and North Interlake herds as a separate community and the walktrap algorithm clustered The Bog and North Interlake as separate communities from Manitoba, as well as each other (Table S1.5). The other three algorithms (edge betweenness, infomap, and label propagation) detected two communities (The Bog and North Interlake and all other sampling locations; Table S1.5). Modularity scores ranged from 0.01 for the edge betweenness, infomap, and label propagation algorithms to 0.12 for the spinglass, fastgreedy, and leading eigenvector algorithms (Table S1.1).

#### 3.5.2 *F*_ST_

The *F*_ST_ network was thinned using a Dpe of 0.013 and revealed only one large component that connected the majority of sampling locations (*n* = 22), whereas the remainder of sampling locations consisted of individual disconnected components (i.e., no edges connecting them to other sampling locations: Ignace, Kenogami, Kississing, North Interlake, and The Bog; Figure 4b). The number of clusters identified by the community detection algorithms ranged from 7 for the label propagation technique to 11 for the walktrap technique and modularity scores ranged from 0.11 for the label propagation technique to 0.26 for the multilevel algorithm (Table S1.1). Similar to *R*_ST_, there was some variability regarding cluster membership across community detection algorithms (Table S1.5). However, all algorithms clustered northwestern Manitoba (Naosap–Reed, Wabowden, Wapisu–Wimapedi, Wheadon) separately from other areas, as well as the five disconnected sampling locations (Table S1.5). Additionally, the three algorithms with the largest modularity scores (multilevel, leading eigenvector, and fastgreedy; Table S1.1) identified three additional clusters that were similar among techniques, including a group in eastern Ontario (Cochrane, Kapuskasing, Moosonee, and Webequie [multilevel and fastgreedy only], and Marten Falls [leading eigenvector and fastgreedy only]), a group primarily found in eastern Manitoba and western Ontario (Atiko, Charron Lake, Norway House, Woodland Caribou Provincial Park, and Attawapiskat [from northern Ontario]), and a group primarily found in Ontario (Big Trout Lake, Keewaywin, Nipigon, Sioux Lookout, Red Lake, Wabakimi, Weagamow, and Berens [from eastern Manitoba]); the multilevel algorithm identified an additional group within the Ontario group in the southern portion of the range (Nipigon, Red Lake, Sioux Lookout, and Wabakimi; Figure 4b).Overall, these results suggested that the *F*_ST_ networks identified more recently isolated groups, particularly in the southern part of the study area.

### 3.6 Recent migration rates

**Figure 5:**
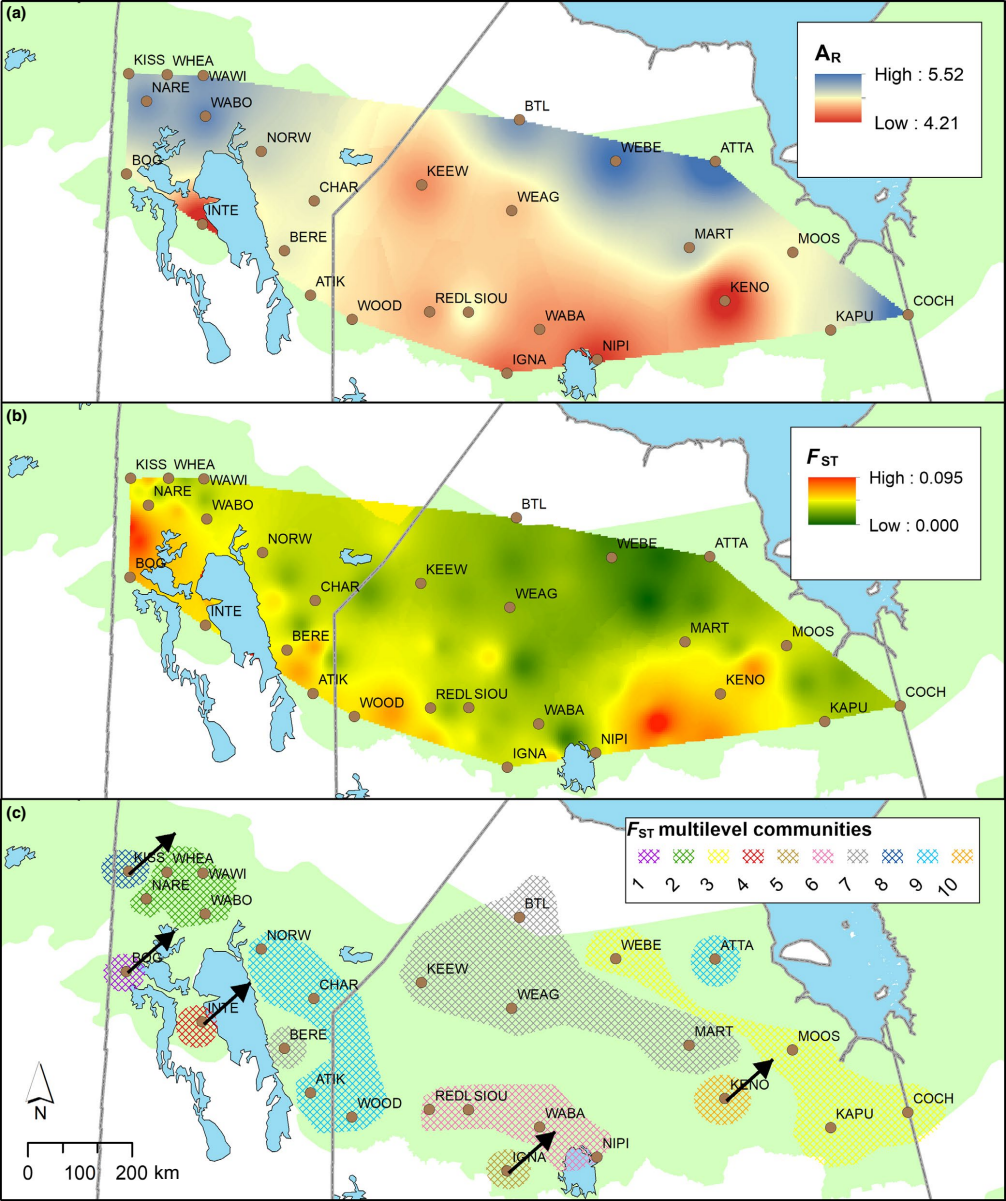
Interpolated surface of (a) genetic diversity (rarefied number of alleles [allelic richness or *A*_R_]) and (b) genetic differentiation (*F*_ST_) across the boreal caribou range in Ontario/Manitoba, Canada. Ancestry to (c) the 10 multilevel communities (based on *F*_ST_) is represented by colors. Genetic groups with migration going to other populations but not acquiring migrants from other populations (asymmetrical gene flow based on the BIMr results) are represented with a black arrow

Recent migration rates calculated in BIMr were based on the 10 groups identified from the multilevel community detection algorithm (Table S1.1) computed for *F*_ST_ instead of the sampling locations; this pooling of groups was necessary because of convergence problems when the population number is >10 (Oscar Gaggiotti, personal communication). Yet, because the 10 groups represent contemporary patterns of genetic structure, we argue that pooling of sampling locations represents the most appropriate delineation for exploring recent migration rates. We chose the multilevel community detection algorithm over other community detection techniques because it produced the largest modularity score (Table S1.1). However, because the *F*_ST_ groupings were similar among different community detection techniques (Table S1.5), we assumed that patterns in migration rates would be similar among the different delineations. Recent migration rates ranged from nearly zero among The Bog (multilevel group 1), North Interlake (multilevel group 4), Ignace (multilevel group 5), Kississing (multilevel group 8), and Kenogami (multilevel group 10) groups (Table 3, Figure 5). Migration rates between those five groups and the remaining groups were asymmetrical, where the proportion of migrant genes from multilevel groups 1, 4, 5, 8, and 10 were detected in greater frequency in the five other multilevel groups (groups 2, 3, 6, 7, and 9), as indicated by nonoverlapping HPDI values, than *vice versa* (Table 3). These results suggested that especially groups at the southern range margin are highly isolated and do not receive/exchange much genetic material from other populations, which is in line with the generally lower genetic diversity estimates found in the current study. Hence, our results indicated that population connectivity at the range margins has fallen below a critical level and negatively affects genetic diversity.

**Table 3:** Mean (top line) and mode (second line) migration rates among caribou groups (based on the 10 multilevel groups identified from *F*_ST_) and lower (third line) and upper (fourth line) HPDI values inferred by the program BIMr. Rows represent the populations from which each individual was sampled, and columns represent the population from which they migrated. Values along the diagonal are the proportion of individuals identified as residents in the source population. Bold text indicates nonoverlapping HPDI values

Into/From	1	2	3	4	5	6	7	8	9	10
1	1.000	**1.9E−09**	**1.9E−09**	2.0E−09	1.9E−09	**1.9E−09**	**1.9E−09**	1.9E−09	**2.0E−09**	1.9E−09
	1.000	3.3E−10	2.0E−10	2.6E−10	2.5E−10	3.3E−10	3.2E−10	2.3E−10	3.7E−10	2.0E−10
	1.000	2.2E−09	3.6E−09	2.4E−09	1.9E−09	9.9E−10	1.2E−09	1.2E−10	6.5E−14	2.0E−10
	1.000	5.6E−08	1.0E−07	6.4E−08	6.3E−08	5.4E−08	5.3E−08	5.6E−08	8.9E−08	9.9E−08
2	**0.172**	0.418	0.056	**0.009**	**0.008**	0.006	0.005	**0.005**	0.286	**0.033**
	0.172	0.418	0.049	0.002	0.002	0.001	9.9E−04	0.001	0.289	0.007
	0.008	0.265	0.008	0.004	0.004	0.002	3.3E−04	0.001	0.126	0.007
	0.330	0.582	0.169	0.075	0.076	0.073	0.054	0.052	0.436	0.234
3	**0.016**	0.019	0.494	**0.370**	**0.024**	0.028	0.012	**0.012**	0.012	**0.013**
	0.003	0.004	0.491	0.364	0.005	0.006	0.002	0.002	0.002	0.003
	0.005	0.003	0.186	0.145	0.004	0.008	0.001	0.004	0.005	0.001
	0.127	0.166	0.745	0.611	0.230	0.208	0.137	0.109	0.123	0.167
4	1.7E−08	**1.8E−08**	**1.7E−08**	1.000	1.8E−08	**1.8E−08**	**1.8E−08**	1.8E−08	**1.8E−08**	1.8E−08
	1.3E−09	1.4E−09	1.3E−09	1.000	2.4E−09	1.3E−09	1.4E−09	1.3E−09	2.4E−09	1.3E−09
	3.2E−08	9.8E−09	3.8E−09	1.000	2.8E−08	1.6E−08	5.5E−09	2.1E−08	2.9E−08	2.2E−08
	6.6E−07	6.9E−07	6.2E−07	1.000	6.2E−07	6.7E−07	6.7E−07	6.5E−07	6.1E−07	6.5E−07
5	6.3E−09	**6.3E−09**	**6.3E−09**	6.2E−09	1.000	**6.3E−09**	**6.3E−09**	6.3E−09	**6.3E−09**	6.3E−09
	1.1E−09	1.1E−09	1.2E−09	1.3E−09	1.000	1.3E−09	1.1E−09	1.2E−09	1.0E−09	1.2E−09
	5.3E−09	8.8E−09	2.0E−09	1.7E−09	1.000	8.0E−09	4.5E−09	8.9E−09	6.9E−10	1.8E−09
	1.9E−07	1.8E−07	2.0E−07	1.6E−07	1.000	2.1E−07	1.9E−07	2.1E−07	1.7E−07	1.5E−07
6	**0.014**	0.019	0.027	**0.149**	**0.240**	0.430	0.062	**0.013**	0.030	**0.016**
	0.003	0.004	0.006	0.142	0.237	0.427	0.049	0.003	0.011	0.003
	0.001	0.001	0.001	0.014	0.039	0.212	0.009	0.005	0.007	0.001
	0.125	0.140	0.193	0.380	0.482	0.683	0.236	0.107	0.164	0.179
7	**0.015**	0.017	0.043	**0.037**	**0.119**	0.055	0.492	**0.165**	0.032	**0.026**
	0.003	0.003	0.028	0.007	0.091	0.043	0.502	0.161	0.023	0.005
	0.004	0.007	0.007	0.003	0.002	0.014	0.203	0.059	0.007	0.007
	0.132	0.158	0.183	0.205	0.389	0.295	0.688	0.303	0.163	0.180
8	1.8E−08	**1.8E−08**	**1.8E−08**	1.8E−08	1.7E−08	**1.8E−08**	**1.8E−08**	1.000	**1.8E−08**	1.8E−08
	1.5E−09	1.7E−09	1.6E−09	1.9E−09	2.8E−09	2.6E−09	1.6E−09	1.000	1.5E−09	2.8E−09
	1.8E−08	2.0E−08	7.8E−09	4.0E−08	2.3E−13	1.7E−08	4.7E−09	1.000	2.3E−08	5.5E−09
	7.5E−07	8.4E−07	7.7E−07	9.6E−07	6.9E−07	6.6E−07	7.6E−07	1.000	7.6E−07	6.8E−07
9	**0.087**	0.042	0.010	**0.022**	**0.035**	0.013	0.014	**0.184**	0.351	**0.241**
	0.081	0.010	0.002	0.004	0.026	0.002	0.003	0.178	0.350	0.237
	0.007	0.006	0.003	0.007	0.003	0.002	0.001	0.076	0.089	0.029
	0.296	0.216	0.106	0.163	0.155	0.149	0.131	0.341	0.596	0.466
10	7.1E−09	**7.2E−09**	**7.2E−09**	7.2E−09	7.2E−09	**7.2E−09**	**7.2E−09**	7.1E−09	**7.2E−09**	1.000
	1.4E−09	1.4E−09	1.5E−09	1.5E−09	1.4E−09	1.4E−09	1.4E−09	1.5E−09	1.4E−09	1.000
	2.3E−09	3.1E−09	1.5E−09	1.0E−09	6.1E−10	1.4E−08	2.7E−09	2.0E−13	2.2E−09	1.000
	7.7E−08	7.8E−08	6.6E−08	7.2E−08	7.4E−08	7.6E−08	8.5E−08	6.5E−08	7.0E−08	1.000

### 3.7 Mapping of genetic erosion

Surfaces of genetic diversity and differentiation (based on *F*_ST_) indicated that erosion was occurring in the southern and western portions of the study area (Figure 5).

## 4 DISCUSSION

Changes in the distribution of species due to undesirable environmental conditions, either through avoidance or local extinction, are likely to alter the genetic connectivity and diversity, which has implications for the species' evolutionary potential (Pauls, Nowak, Bálint, & Pfenninger, 2013). Consequences of shifting species' distributions and subsequent genetic erosion can reduce fitness (Leroy et al., 2018) and severely limit evolutionary responses under stressful environmental conditions (Bijlsma & Loeschcke, 2012). Our study provides insights on spatial genetic diversity, genetic connectivity, and movement patterns of boreal caribou in the contiguous portions of their range in Ontario/Manitoba, a distribution that has retracted substantially during the last century (Schaefer, 2003; Vors, Schaefer, Pond, Rodgers, & Patterson, 2007). Assessing genetic erosion in this species is important for identifying areas of management focus, particularly where the monitoring of demographic parameters may be costly and difficult (Leroy et al., 2018). Additionally, boreal caribou play important ecological roles in the boreal forest from nutrient cycling to providing a food source for a variety of predators (Hummel & Ray, 2008; Vors & Boyce, 2009). Therefore, understanding impacts of anthropogenic changes on their populations may provide insights on the vulnerability of other species in the boreal forest, as well as species that are threatened by global change in general.

We used a unique combination of spatial and temporal approaches to differentiate between contemporary and historical patterns of genetic structure. Although a variety of metrics have been proposed for assessing genetic erosion (Hoban et al., 2014; Leroy et al., 2018), our use of networks with measures of genetic differentiation as edge distances showed to be a powerful approach to understand the patterns of genetic connectivity. The results support our predictions that contemporary patterns of genetic differentiation would differ from historical patterns, potentially as a result of the prevalence of anthropogenic activity in the southern part of the range. The *F*_ST_ network suggested populations found along the southern range margin, in particular, exhibited differentiation from interior groups. Isolation by distance was not likely a major factor driving contemporary patterns of genetic structure patterns, differing from the findings of Priadka et al. (2018). However, we explored isolation by distance at the level of sampling locations and clusters, whereas Priadka et al. (2018) focused on the individual level. Instead, recent landscape changes may be underlying factors responsible for generating recent genetic structure at the scale we focused on (as opposed to isolation by distance). Previous research has shown that anthropogenic features can negatively impact boreal caribou through direct habitat loss (Galpern, Manseau, & Wilson, 2012; Schaefer, 2003). Additionally, linear features, including roads, trails, and power lines, may facilitate the movement of predators and increase access to caribou habitat and the potential for caribou mortality(COSEWIC, 2011; James & Stuart‐Smith, 2000). Moreover, forestry practices, including forest cutovers, may also impact caribou populations by changing the composition and configuration of the forest, including a loss of old‐growth pine (*Pinus* spp.) and spruce (*Picea* spp.) forests (Vors et al., 2007). Forest cutovers can alter caribou food supplies and increase moose and deer numbers, which also increases predator numbers, all of which potentially contribute to reduced population sizes and increased genetic erosion in caribou at the southern distribution margins.

Our findings were similar to Priadka et al. (2018) in that we detected contemporary genetic structure in western Manitoba. Although this region is not located near the range margin, anthropogenic activities are greater in those regions compared with the more northern regions of the study area (Environment Canada, 2011). Additionally, natural fragmentation associated with large lakes results in a more patchy distribution of preferred habitat (Fall, Fortin, Manseau, & O'Brien, 2007). Kurz and Apps (1999) showed that fire was the primary disturbance factor west of the Manitoba–Ontario border, burning nine times more forest area within the last 40 years than east of that border. It has also been suggested that wolf densities are greater in the southern and western portions of the boreal forest (i.e., central Manitoba; Darby et al., 1989) where the occurrence of fire is also prevalent. Caribou in those areas tend to select peat bogs surrounded by coniferous forest (Koper & Manseau, 2009; O'Brien, Manseau, Fall, & Fortin, 2006) to provide some separation from predators. It is important to note, however, that the impacts of fire and predators are not likely to be recent factors only. Caribou have evolved with fire and predators (Musiani et al., 2007) and those factors may have impacts on both historical and contemporary genetic structure. Therefore, careful examination of potential underlying factors is warranted to distinguish between natural and human‐induced impacts on genetic diversity.

Contemporary genetic differentiation was lower in northern portions of Ontario. Although anthropogenic activity is less widespread in that region and therefore one possible explanation for this pattern, the eastern migratory caribou (an ecotype that is migratory or where the summer and winter ranges do not overlap; Figure 2), found in northern Ontario and northeastern Manitoba, may also play a role in contemporary connectivity patterns. Telemetry records have shown that eastern migratory animals overlap seasonally with boreal animals in Ontario (Berglund et al., 2014; Pond, Brown, Wilson, & Schaefer, 2016). Consequently, that ecotype potentially interbreeds with boreal animals and their greater mobility likely increases gene flow within and among boreal caribou sampled areas (Thompson, 2015), which is similar to findings in Quebec (Boulet, Couturier, Côté, Otto, & Bernatchez, 2007). Our network analysis allowed for the calculation of node metrics, which can be used to identify hubs of connectivity and prioritize areas for management (Cross et al., 2018; Garroway et al., 2008; Koen et al., 2016). We found that most node metrics based on *F*_ST_ suggested that sampled areas in northern regions of the Ontario boreal range may be important for supplementing southernmost populations (see Appendix S1.5, Table S1.4). The clear spatial difference between connectivity in northern versus southern regions calculated in the *F*_ST_ network further suggested that southern populations were more isolated from one another in recent time frames. Therefore, even if the eastern migratory caribou potentially contributed to connectivity in northern regions in the study area, this cannot sufficiently counteract isolation effects in southern parts.

Historical differentiation contributed to genetic structure of boreal caribou in Ontario and Manitoba, but at the level of STRUCTURE and regional and local TESS clusters. This was supported by pairwise tests evaluating the effect of mutations on genetic structure, which were significant when caribou individuals were grouped by the TESS regional and local clusters. Similar to contemporary causes of structure, it is unlikely that the historical structure is the result of isolation by distance as shown by our analyses. Instead, three lines of evidence (i.e., pairwise tests, community detection techniques, and AMOVA for *R*_ST_) suggested that some of the differentiation may be the result of phylogeographic processes. One possibility is that the eastern migratory ecotype, which most likely diverged after an earlier introgression between barren‐ground caribou (*Rangifer tarandus groenlandicus*; Figure 2) and a woodland caribou lineage (Klütsch et al., 2016), might have been a conduit for the transfer of different genetic material into boreal caribou and generating a signal of genetic structure between western Manitoba and eastern Manitoba/Ontario. Indeed, a greater number of barren‐ground mtDNA haplotypes were found in the boreal range of eastern Manitoba/Ontario when compared to western Manitoba (Klütsch et al., 2016, 2012).

Alternatively, vicariance may explain the historical structure patterns seen. The mitochondrial DNA study by Klütsch et al. (2012) found that while both Manitoba and Ontario animals expanded from refugia found south of the Laurentide ice sheet after the last glacial maximum, the Ontario animals likely expanded from a refugium in the Appalachian Mountains, while Manitoba animals likely expanded from a refugium found west of the Appalachian Mountains and east of the Mississippi River (Klütsch et al., 2012). For example, Klütsch et al. (2016) also delineated an Ontario and eastern Manitoba group, a western Manitoba group, and a southwestern Manitoba group and showed that many of those broad‐level patterns corresponded with the proportion of ancestry from different woodland caribou lineages. The majority of community detection algorithms used to analyze the *R*_ST_ network identified similar patterns as our Bayesian clustering results, delineating an Ontario group and either a Manitoba group or western and southwestern Manitoba groups (Table S1.5). However, 5 of the 8 algorithms clustered some eastern Ontario sampled areas with the Manitoba groups (i.e., Attawapiskat, Big Trout Lake, Marten Falls, and Webequie; Table S1.5). Additionally, some eastern Manitoba and western Ontario groups also clustered with the western Manitoba group in 4 of the 8 algorithms (Table S1.5). Klütsch et al. (2016) detected small proportion of haplotypes from the western Manitoba haplogroup in some of those eastern Manitoba and Ontario sampled areas, which may explain why several of the community detection algorithms clustered them similarly. Hence, the current study is consistent with findings from previous studies in that broad‐scale genetic differentiation is evidently the result of historical/ancient differentiation processes.

Similar to genetic differentiation, there were associations between genetic diversity and distance to the southern range margin (Table 1), an area where anthropogenic activity is greater than in other portions of the study area. Additionally, although there was not an association between assortative mating and distance to the southern range margin, larger *F*_IS_ values were found in some sampled areas within 100 km of the range's southern edge (e.g., Atiko and Nipigon; Table 1), suggesting potentially smaller effective population sizes in those areas (Wang, Santiago, & Caballero, 2016). It is important to note, however, that although we removed locations with sample sizes <10 individuals and some of our genetic diversity estimates accounted for differences in sample sizes, other factors may bias results. For example, Goldberg and Waits (2010) showed that sampling closely related individuals (e.g., siblings) may impact estimates of both genetic diversity and differentiation.

Evidence of genetic bottlenecks was found only in the Kenogami area and only for small prebottleneck effective population sizes (Table S1.2). However, the power of the tests to detect small declines can be low, particularly when modest sample sizes or the number of loci is small or when sampling a short amount of time after a bottleneck has occurred (Peery et al., 2012). The Kenogami area is found close to the southern periphery of the range and may explain the bottleneck signal (Figure 2); for the *M*‐ratio test, we assumed a smaller proportion of one‐step mutations (*p*_s_ = 0.80; see Appendix S1.2) than is recommended by Garza and Williamson (2001), which may minimize the likelihood of a type I error, assuming the true proportion is not exceptionally small (Peery et al., 2012).

Our results also supported our final prediction that recent migration rates, as inferred through recent proportions of immigrant genes in subdivided populations, would show a movement pattern away from the southern range limit. These results are consistent with animals emigrating from lower quality habitats (Andreasen, Stewart, Longland, Beckmann, & Forister, 2012). The Kississing was the only sampling location distant from the southern range limit that exhibited asymmetrical gene flow. However, anthropogenic activity is again greater in that region than in the northern parts of the distribution. The estimates represented migration rates from within the last generation (Faubet & Gaggiotti, 2008) and may suggest a continuing retraction of the boreal caribou range in Ontario and Manitoba, with potentially greater penetration into western Manitoba.

## 5 CONCLUSIONS

Our findings illustrate recent genetic erosion and increased inbreeding as well as decreased connectivity in the contiguous southern distribution of boreal caribou in Ontario and Manitoba suggesting ongoing range retraction. A northward migration trend suggests that animals partially react with avoidance to disturbed areas. These findings are consistent between two regional differences in population history: Ontario into the east side on Lake Winnipeg and the remaining distribution of Manitoba. These regions demonstrate different histories that contribute to different levels of baseline genetic structure and contact with the eastern migratory ecotype, yet the signatures of genetic erosion are consistently evident. As a result, our study demonstrates that fine‐scale genetic analysis, when accounting for historical processes, is valuable to assess the impact of human‐induced landscape changes on genetic diversity and connectivity in wildlife species.

Our research shows provincial and federal recovery efforts would best be focused in areas with healthy and sustainable populations to either restore connectivity among herds or ensure that existing connectivity is maintained. Further, this work has implications for conservation and land protection, particularly for caribou groups that may be important for contemporary gene flow. Thus, consideration of impacts to caribou connectivity and the protection of distinct genetic biodiversity is essential when proposing development plans.

## CONFLICT OF INTEREST

None declared.

## Data Availability

The data used in the current study are a subset of a previously assembled data set which is deposited on Dryad. Data package: Klütsch CFC, Manseau M, Trim V, Polfus J, Wilson PJ (2016) Data from: The eastern migratory caribou: the role of genetic introgression in ecotype evolution. Dryad Digital Repository. http://dx.doi.org/10.5061/dryad.4v0d4.
