# Supplementary material for: Spatial differences in genetic diversity and northward migration suggest genetic erosion along the boreal caribou southern range limit and continued range retraction
Source: Ecol Evol. 2019 May 26;9(12):7030–46. doi: 10.1002/ece3.5269 (PMC6662424; doi:10.1002/ece3.5269)
Supplement: Supplementary file 1 [file ECE3-9-7030-s001.docx]

**Spatial differences in genetic diversity and northward migration suggest genetic erosion along the boreal caribou southern range limit and continued range retraction**

**Appendix S1**

**S1.1- Description of ecozones in study area**

The Hudson Plains ecozone covers northern Ontario and extends into northeastern Manitoba and western Quebec. It is characterized by extensive wetlands and coastal marshes with vegetation ranging from arctic tundra to boreal forest transition types (Ecological Stratification Working Group, 1995). The boreal shield is Canada’s largest ecozone and consists of large regions of coniferous forests interspersed by exposed bedrock. A large number of lakes are also present (Ecological Stratification Working Group, 1995). The boreal plains ecozone is located to the south and west of the boreal shield and wetland and peat bog areas make up as much as 25-50% of this region (Ecological Stratification Working Group, 1995). Both the boreal plain and shield can be characterized by frequent forest fire; however, natural fire occurrence generally decreases from the southern to the northern limits of the boreal forest and from west to east (Johnson, 1992). In general, the amount of disturbance is least in the Far North regions of Ontario and greatest in regions found along the current southern range margin in Ontario and Manitoba and in northwestern regions of Manitoba (Environment Canada, 2012).

**S1.2- Background and run parameters for detection of genetic bottlenecks**

BOTTLENECK (v1.2.02; Piry et al. 1999) assumes that allelic diversity is lost at a faster rate than heterozygosity and, therefore, tests for an excess of heterozygosity compared to what would be expected at mutation-drift equilibrium (Cornuet & Luikart, 1996). Deviations were inferred using a Wilcoxon sign-rank test with 1000 iterations under two models: stepwise mutation model (SMM) and two-phase mutation model (TPM). The TPM was evaluated under a range of parameters where the proportions of mutations that followed a single step were 0.70, 0.80, and 0.95 and the variances of multiple steps were 30 and 12. Because this test may only detect bottlenecks within the last ~10 generations (Peery et al., 2012), we also used the M-ratio statistic, or the total number of alleles divided by the range in allele size, to test for reductions in population size (Garza & Williamson, 2001). This test assumes that the number of alleles is going to decrease faster than the range of allele sizes, producing a smaller ratio in recently reduced populations, and has the potential to detect population declines between 10-50 generations or longer (Garza & Williamson, 2001). We calculated the observed ratio for each sampled area using program M P VAL (version 1.0; Garza and Williamson 2001). Additionally, we calculated the critical value for M (M_c_), which is the point where a past reduction in population size can be inferred given a specific range of parameters, using CRITICAL M (version 1.0; Garza and Williamson 2001). These parameters include Θ (or 4N_e_µ, where N_e_ is the effective population size prior to a bottleneck event and µ is the mutation rate), p_s_ (the proportion of one-step mutations), and Δ_g_ (the average size of non one-step mutations; Garza and Williamson 2001). We calculated M_c_ using p_s_ = 0.8, Δ_g_ = 3.5, and a range of Θ values, where N_e_ = 50, 100, 1000, and 1500 and µ = 1.5 x 10^-3^ and 1.5 x 10^-4^.

**S1.3- Explanation of node metrics used in network approach**

*Degree* is the number of edges connected to each node and represents the level of connectivity of a particular node (Kivelä et al., 2015). *Betweenness centrality* is the number of shortest paths passing through a node that also pass through other nodes (Freeman, 1977) and measures how important a particular region is for maintaining gene flow (Kivelä et al., 2015). The *clustering coefficient* (or transitivity measure) is the ratio of connected node triplets compared to the possible number of node triplets in the network (Watts & Strogatz, 1998; Girvan & Newman, 2002). A high average clustering coefficient over all nodes suggests that a network is non-random and has hierarchical structure (Kivelä et al., 2015). Koen et al. 2016) used simulations to show that average inverse edge weight (the inverse of edge weight averaged across edges for each node; AIEW) exhibited a linear relationship with connectivity and may be a better node-based measure than degree or betweenness centrality.

Table S1.1: Community detection algorithm definitions, citations, and number of boreal caribou communities and associated modularity scores calculated based on each algorithm for R_ST_ and F_ST_.

|  |  |  | **R_ST_** | | **F_ST_** | |
| --- | --- | --- | --- | --- | --- | --- |
| **Community detection algorithm** | **Algorithm description** | **Source** | **Number of modules** | **Modularity** | **Number of modules** | **Modularity** |
| Spinglass^*^ | Based on a physics model where each node can be in a set of spin states and the interactions between nodes (edges) can determine which nodes stay in the same spin state | Reichardt and Bornholdt 2006 | 3 | 0.12 | NA | NA |
| Fastgreedy | A hierarchical approach that seeks to optimize modularity by assuming that every node belongs to a different community, which are subsequently merged to determine which community groups yield the largest modularity | Clauset et al. 2004 | 2 | 0.12 | 9 | 0.25 |
| Leading eigenvector | A bottom-up hierarchical approach that seeks to optimize modularity by assuming a connected graph that is subsequently split and determines which groups yield the largest modularity | Newman 2006 | 2 | 0.12 | 9 | 0.24 |
| Multilevel | A hierarchical approach that seeks to optimize modularity by assuming that every node belongs to a different community and are then merged with local communities through a series of steps; the node is then assigned to the community that produces the largest modularity; this process continues until no more nodes can be re-assigned | Blondel et al. 2008 | 4 | 0.11 | 10 | 0.26 |
| Walktrap | A random-walk approach that identifies communities by assuming that a node will stay in the community it belongs because of a smaller number of edges that connect it to other communities | Pons and Latapy 2005 | 5 | 0.09 | 11 | 0.17 |
| Edge betweenness | A top-down hierarchical approach that removes edges in decreasing order of edge betweenness values | Girvan and Newman 2002 | 2 | 0.01 | 8 | 0.14 |
| Infomap | An information theoretic approach that uses the probability of the flow of random walks as a proxy for information flow throughout the network and reduces probability flows, generating network modules | Rosvall and Bergstrom 2007, Rosvall et al. 2009 | 2 | 0.01 | 8 | 0.14 |
| Label propagation | Assigns nodes to unique "labels" and then nodes are reassigned labels so that they take labels close to those of their neighbors | Raghavan et al. 2007 | 2 | 0.01 | 7 | 0.11 |

^*^The spinglass community detection algorithm cannot delineate communities in networks with disconnected nodes and was, therefore, not computed for the F_ST_ network.

Table S1.2: Tests for genetic bottlenecks based on heterozygosity access and M-ratio values. The tests based on heterozygosity access were conducted using the stepwise mutation (SMM) and two-phase mutation models (TPM), the latter of which were conducted under a range of mutation proportions that do not follow a single step and variances. Observed M-ratios were calculated for each sampled area and corresponding critical M values (Mc) were calculated across a range of theta values.

|  | | BOTTLENECK | | | | |  | | M-ratio test | | | | | | | | |
| --- | --- | --- | --- | --- | --- | --- | --- | --- | --- | --- | --- | --- | --- | --- | --- | --- | --- |
| Sampled area | SMM | | TPM_70_30 | TPM_95_30 | TPM_95_12 | TPM_80_12 | | | | Observed M | Mc (theta=0.03) | Mc (theta=0.3) | Mc (theta=0.6) | Mc (theta=0.9) | Mc (theta=3.0) | Mc (theta=6.0) | Mc (theta=9.0) |
| WOOD | 0.999 | | 0.990 | 0.998 | 0.999 | 0.995 | |  | | 1.155 | 0.770 | 0.739 | 0.711 | 0.692 | 0.644 | 0.637 | 0.632 |
| REDL | 1.000 | | 0.898 | 0.997 | 0.999 | 0.976 | |  | | 1.624 | 0.767 | 0.740 | 0.708 | 0.690 | 0.644 | 0.630 | 0.628 |
| SIOU | 1.000 | | 0.995 | 1.000 | 1.000 | 0.999 | |  | | 1.309 | 0.769 | 0.732 | 0.712 | 0.689 | 0.640 | 0.625 | 0.617 |
| IGNA | 0.787 | | 0.590 | 0.752 | 0.752 | 0.715 | |  | | 1.058 | 0.769 | 0.734 | 0.707 | 0.680 | 0.594 | 0.546 | 0.518 |
| WABA | 0.986 | | 0.180 | 0.875 | 0.918 | 0.545 | |  | | 1.170 | 0.773 | 0.735 | 0.709 | 0.686 | 0.623 | 0.598 | 0.584 |
| NIPI | 0.976 | | 0.500 | 0.850 | 0.898 | 0.752 | |  | | 1.019 | 0.767 | 0.735 | 0.708 | 0.683 | 0.609 | 0.570 | 0.548 |
| KEEW | 0.993 | | 0.674 | 0.787 | 0.875 | 0.715 | |  | | 0.833 | 0.769 | 0.734 | 0.707 | 0.687 | 0.627 | 0.607 | 0.596 |
| WEAG | 0.976 | | 0.674 | 0.918 | 0.936 | 0.850 | |  | | 1.144 | 0.770 | 0.733 | 0.706 | 0.679 | 0.603 | 0.567 | 0.543 |
| BTL | 0.999 | | 0.986 | 0.997 | 0.997 | 0.993 | |  | | 1.366 | 0.769 | 0.737 | 0.708 | 0.689 | 0.633 | 0.617 | 0.606 |
| WEBE | 0.986 | | 0.715 | 0.936 | 0.976 | 0.875 | |  | | 1.389 | 0.769 | 0.736 | 0.703 | 0.685 | 0.609 | 0.577 | 0.561 |
| ATTA | 0.850 | | 0.326 | 0.752 | 0.752 | 0.715 | |  | | 1.242 | 0.770 | 0.733 | 0.706 | 0.679 | 0.603 | 0.567 | 0.543 |
| MART | 0.998 | | 0.898 | 0.995 | 0.997 | 0.976 | |  | | 1.285 | 0.769 | 0.738 | 0.705 | 0.685 | 0.619 | 0.591 | 0.575 |
| MOOS | 0.993 | | 0.820 | 0.981 | 0.981 | 0.918 | |  | | 0.995 | 0.768 | 0.738 | 0.711 | 0.685 | 0.626 | 0.601 | 0.591 |
| KENO | 0.998 | | 0.997 | 0.998 | 0.998 | 0.998 | |  | | 0.726 | **0.769** | **0.734** | 0.707 | 0.680 | 0.594 | 0.546 | 0.518 |
| KAPU | 0.998 | | 0.918 | 0.995 | 0.998 | 0.990 | |  | | 1.226 | 0.767 | 0.735 | 0.708 | 0.683 | 0.609 | 0.570 | 0.548 |
| COCH | 0.986 | | 0.367 | 0.918 | 0.918 | 0.752 | |  | | 1.277 | 0.769 | 0.734 | 0.707 | 0.687 | 0.627 | 0.607 | 0.596 |
| KISS | 0.981 | | 0.125 | 0.674 | 0.715 | 0.326 | |  | | 1.253 | 0.767 | 0.735 | 0.710 | 0.690 | 0.648 | 0.645 | 0.642 |
| NARE | 0.993 | | 0.633 | 0.936 | 0.981 | 0.875 | |  | | 1.172 | 0.770 | 0.737 | 0.712 | 0.693 | 0.663 | 0.666 | 0.672 |
| WHEA | 0.976 | | 0.500 | 0.850 | 0.898 | 0.590 | |  | | 1.215 | 0.767 | 0.741 | 0.709 | 0.691 | 0.644 | 0.635 | 0.632 |
| WAWI | 0.993 | | 0.898 | 0.990 | 0.990 | 0.976 | |  | | 1.345 | 0.772 | 0.738 | 0.711 | 0.691 | 0.662 | 0.667 | 0.666 |
| WABO | 0.999 | | 0.875 | 0.990 | 0.997 | 0.976 | |  | | 1.248 | 0.769 | 0.736 | 0.711 | 0.693 | 0.656 | 0.653 | 0.655 |
| BOG | 0.995 | | 0.633 | 0.990 | 0.993 | 0.898 | |  | | 0.976 | 0.769 | 0.735 | 0.709 | 0.690 | 0.650 | 0.640 | 0.641 |
| INTE | 1.000 | | 0.285 | 0.367 | 0.633 | 0.285 | |  | | 0.921 | 0.769 | 0.736 | 0.711 | 0.693 | 0.656 | 0.653 | 0.655 |
| NORW | 0.997 | | 0.918 | 0.986 | 0.990 | 0.981 | |  | | 1.134 | 0.773 | 0.735 | 0.709 | 0.686 | 0.623 | 0.598 | 0.584 |
| BERE | 0.995 | | 0.752 | 0.981 | 0.990 | 0.898 | |  | | 1.145 | 0.767 | 0.740 | 0.708 | 0.690 | 0.644 | 0.630 | 0.628 |
| CHAR | 1.000 | | 0.976 | 1.000 | 0.999 | 0.993 | |  | | 1.252 | 0.772 | 0.737 | 0.708 | 0.686 | 0.620 | 0.595 | 0.581 |
| ATIK | 0.997 | | 0.752 | 0.993 | 0.997 | 0.976 | |  | | 1.333 | 0.769 | 0.738 | 0.705 | 0.685 | 0.619 | 0.591 | 0.575 |

**S1.4- Detailed description of STRUCTURE and TESS results**

The *ΔK* values calculated from program STRUCTURE suggested that the highest order of structure was *K* = 2 when caribou individuals from Manitoba and Ontario were pooled; however, the likelihood values continued to increase, suggesting the presence of additional structure (Fig. S1.1a). The model for *K* = 2 suggested some differentiation between Manitoba (northern Manitoba, in particular) and Ontario (Fig. S1.2a). However, all regions were highly admixed, with the exception to southwestern Manitoba (i.e., North Interlake; Fig. S1.2a). When confining our analysis to Manitoba, the Δ*K* peaked at *K* = 3 (Fig. S1.1b) and delineated a northern Manitoba group, a southwestern Manitoba group, and a southeastern Manitoba group (Fig. A2b). However, like the model from both provinces combined, there was a large amount of admixture in northern Manitoba, particularly northeastern Manitoba (Fig. S1.2b). Additionally, some portions of southwestern Manitoba (individuals from The Bog region) exhibited admixture (Fig. S1.2b). When confining our analysis to Ontario, the likelihood values decreased after *K* = 1 and although the *ΔK* values peaked at *K* = 2 (Fig. S1.1c), reviewing the bar plots for *K*>1 suggested a lack of genetic structure in the province (Fig. S1.2c).

The DIC values from the no-admixture model in TESS leveled off abruptly at *K*max = 3 when individuals from Manitoba and Ontario were pooled; the second order rate of change in DIC values also suggested the most likely number of groups was *K*max = 3 (Fig. S1.3a). The admixture values calculated from the BYM model suggested individuals were assigned to a northwestern Manitoba group (Reed, Kississing, Wheadon, Wimapedi, Wabowden, and Naosap; TESS regional cluster 1 or TRC1), a southwestern Manitoba group (The Bog and North Interlake; TRC2), and an eastern Manitoba (Norway House, Charon Lake, Berens, Atiko, and Owl-Flintstone) and Ontario group (TRC3; Fig. 2). When confining our analyses to caribou sampled in Manitoba, the DIC values from the no-admixture model began to level off between *K*max values of 3 and 4 (Fig. S1.3b). However, the second order rate of change in DIC values suggested the most likely number of groups was *K*max = 3 (local clusters; Fig. S1.3b). The admixture model for *K*max = 3 assigned Manitoba individuals similarly to the model when both provinces were combined, including a northwestern Manitoba group (TESS local cluster 1 or TLC1), a southwestern Manitoba group (TLC2), and an eastern Manitoba group (TLC3; Fig. 2b). That model also suggested a large proportion of admixed individuals in the eastern portion of TLC1, western portion of TLC3, and northern portion of TLC2 (Fig. 2b). When confining our analysis to Ontario, the DIC values from the no-admixture model decreased gradually between *K*max = 2 and *K*max = 10 and did not show a clear leveling off (Fig. S1.3c). The second order rate of change in DIC values suggested that the most likely number of groups was *K*max = 3 (Fig. S1.3c). However, the maximum number of clusters revealed by the admixture model (after runs at varying *K*max values) was 2. Because program TESS uses *K*max = 2 as a starting value for potential number of clusters to be inferred, the smallest number of clusters that can be identified by the second order rate of change in DIC is *K*max = 3. Therefore, we assumed that the algorithm would have identified a peak in the second order rate of change at *K*max = 2 if it were possible. The model assigned individuals to a large group in Ontario (we labeled this cluster TLC3 because the regional model did not indicate that Ontario was separate from eastern Manitoba), and a smaller group in eastern Ontario (Cochrane, Kapuskasing; TLC4; Fig. 2c). A large portion of individuals sampled in northern Ontario (the northern portion of TLC3) were admixed (Fig. 2c).


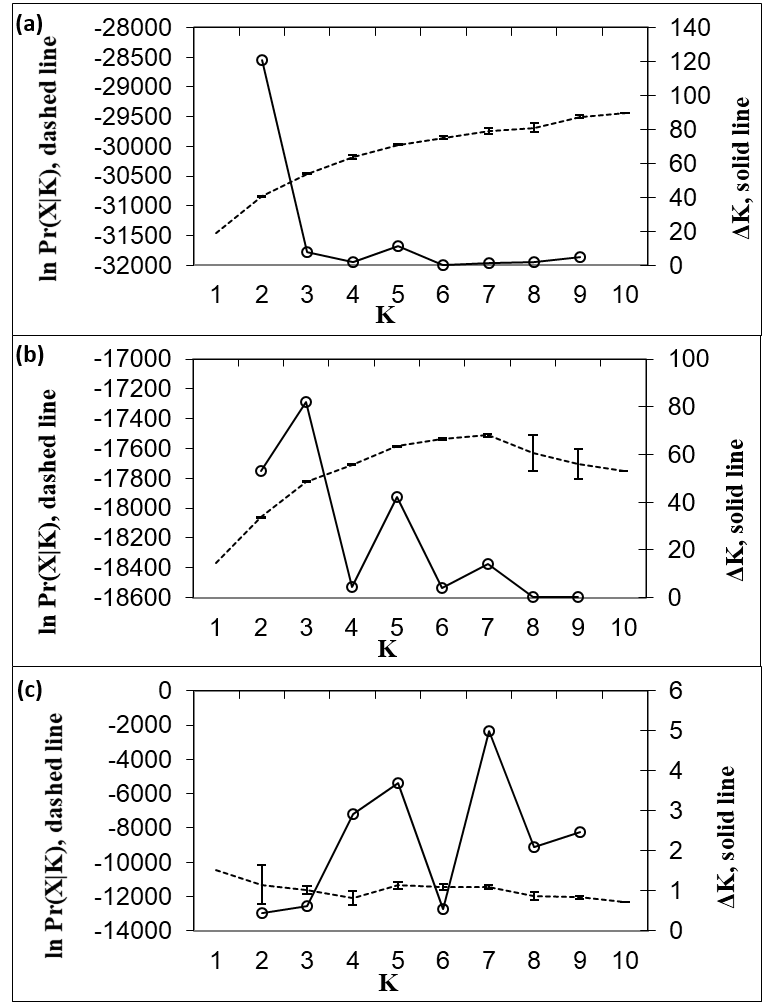


Fig. S1.1. Average likelihood values (lnP(*X*|*K*), dashed line, left y-axis) and the second order rate of change of lnP(*X*|*K*) calculated based on Evanno et al. (2005; solid line, right y-axis) plotted against the number of boreal caribou groups from the five runs in program STRUCTURE. Plots were generated for the complete data set (a) and the two provinces separately (b = Manitoba; c = Ontario).


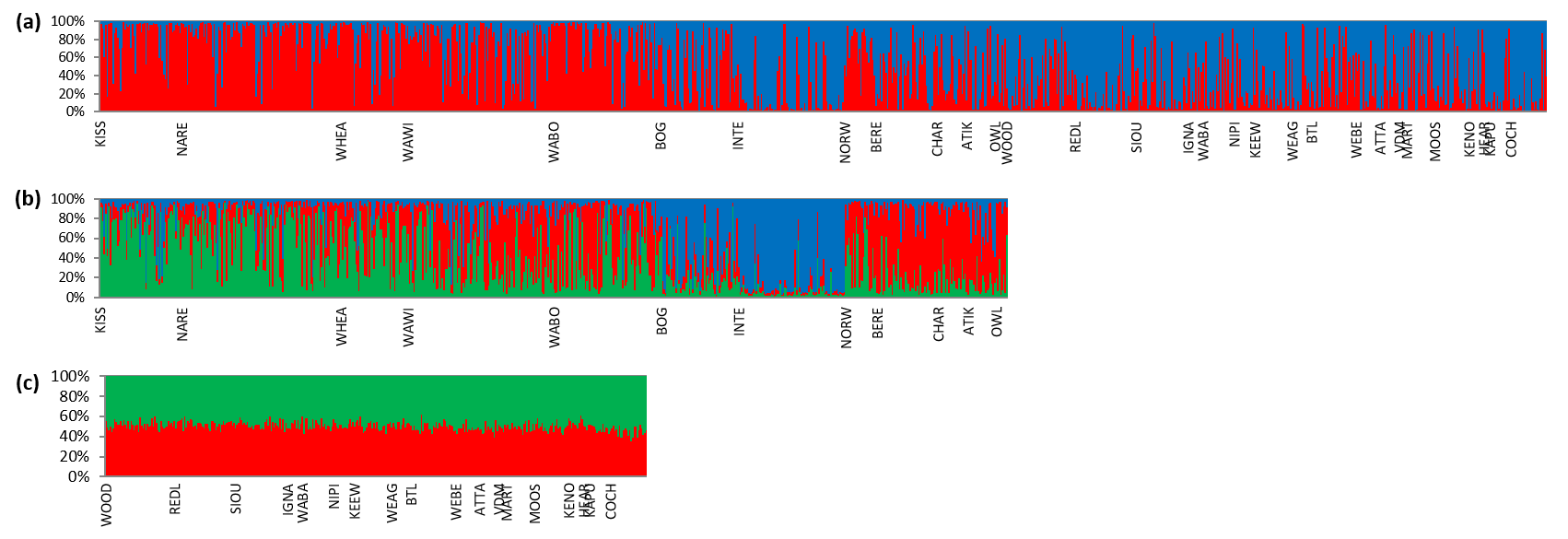


Fig. S1.2. Structure bar plots for boreal caribou for (a) both provinces combined (*K* = 2), (b) Manitoba (*K* = 3), and (c) Ontario (*K* = 2). Each individual caribou is represented by a single vertical bar separated into multiple colored segments with respective lengths proportional to the assignment values of each inferred cluster (*K*). The abbreviated codes on the x-axes correspond to the following sampled areas: Atiko = ATIK, Attawapiskat = ATTA, Berens = BERE, The Bog = BOG, Big Trout Lake = BTL, Charon Lake = CHAR, Cochrane = COCH, Hearst = HEAR, Ignace = IGNA, Kapuskasing = KAPU, Keewaywin = KEEW, Kenogami = KENO, Kississing = KISS, Marten Falls = MART, Moosonee = MOOS, Naosap-Reed = NARE, Nipigon = NIPI, North Interlake = INTE, Norway House = NORW, Owl-Flintstone = OWL, Red Lake = REDL, Sioux Lookout = SIOU, Victor Diamond Mine = VDM, Wabakimi = WABA, Wabowden = WABO, Wapisu-Wimapedi = WAWI, Weagamow = WEAG, Webequie = WEBE, Wheadon = WHEA, Woodland Caribou Provincial Park = WOOD.


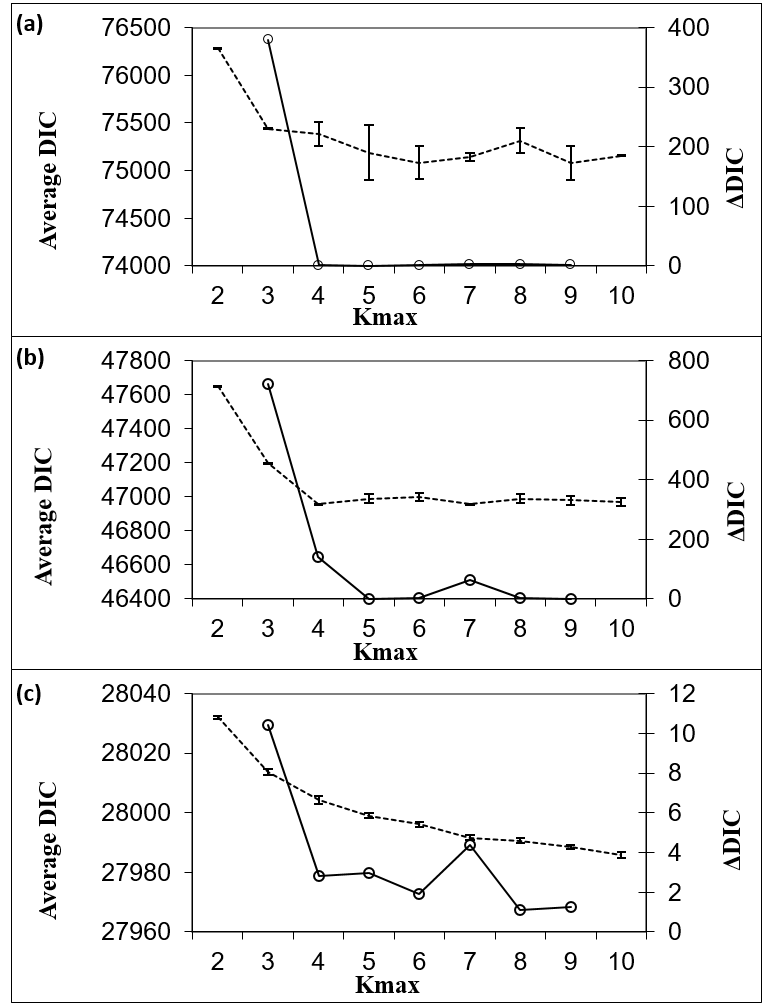


Fig. S1.3. Average DIC values (dashed line, left y-axis) and the second order rate of change of DIC calculated based on Evanno et al. (2005; solid line, right y-axis) plotted against the number of boreal caribou groups (*K*max) from the top 5% of no-admixture model runs in program TESS. Plots were generated for the complete data set (a) and the two provinces separately (b = Manitoba; c = Ontario).

Table S1.3. Pairwise multi-loci R_ST_ estimates (below diagonal) and pairwise multi-loci F_ST_ estimates (above diagonal) for the boreal caribou a) sampled areas, b) TESS regional clusters (TRC), and c) TESS local clusters (TLC).

(a)

|  | WOOD | REDL | SIOU | IGNA | WABA | NIPI | KEEW | WEAG | BTL | WEBE | ATTA | MART |
| --- | --- | --- | --- | --- | --- | --- | --- | --- | --- | --- | --- | --- |
| WOOD | 0.000 | 0.039 | 0.019 | 0.033 | 0.018 | 0.047 | 0.022 | 0.021 | 0.026 | 0.013 | 0.013 | 0.026 |
| REDL | 0.000 | 0.000 | 0.011 | 0.023 | 0.034 | 0.032 | 0.023 | 0.045 | 0.030 | 0.023 | 0.039 | 0.025 |
| SIOU | 0.000 | 0.000 | 0.000 | 0.015 | 0.012 | 0.025 | 0.012 | 0.026 | 0.029 | 0.014 | 0.023 | 0.023 |
| IGNA | 0.000 | 0.001 | 0.005 | 0.000 | 0.020 | 0.031 | 0.030 | 0.049 | 0.044 | 0.030 | 0.037 | 0.044 |
| WABA | 0.000 | 0.000 | 0.001 | 0.000 | 0.000 | 0.000 | 0.012 | 0.000 | 0.024 | 0.007 | 0.018 | 0.018 |
| NIPI | 0.012 | 0.014 | 0.026 | 0.002 | 0.000 | 0.000 | 0.027 | 0.023 | 0.025 | 0.037 | 0.044 | 0.033 |
| KEEW | 0.001 | 0.010 | 0.007 | 0.000 | 0.000 | 0.031 | 0.000 | 0.009 | 0.011 | 0.004 | 0.010 | 0.007 |
| WEAG | 0.000 | 0.002 | 0.011 | 0.000 | 0.000 | 0.011 | 0.005 | 0.000 | 0.003 | 0.012 | 0.008 | 0.011 |
| BTL | 0.000 | 0.000 | 0.000 | 0.000 | 0.000 | 0.014 | 0.012 | 0.017 | 0.000 | 0.010 | 0.000 | 0.003 |
| WEBE | 0.000 | 0.004 | 0.000 | 0.047 | 0.005 | 0.055 | 0.023 | 0.027 | 0.001 | 0.000 | 0.000 | 0.000 |
| ATTA | 0.000 | 0.023 | 0.000 | 0.038 | 0.014 | 0.070 | 0.028 | 0.043 | 0.004 | 0.000 | 0.000 | 0.001 |
| MART | 0.000 | 0.021 | 0.003 | 0.048 | 0.013 | 0.065 | 0.026 | 0.039 | 0.000 | 0.000 | 0.000 | 0.000 |
| MOOS | 0.001 | 0.019 | 0.016 | 0.000 | 0.000 | 0.007 | 0.006 | 0.000 | 0.000 | 0.006 | 0.007 | 0.000 |
| KENO | 0.017 | 0.039 | 0.037 | 0.049 | 0.000 | 0.012 | 0.031 | 0.006 | 0.062 | 0.091 | 0.080 | 0.085 |
| KAPU | 0.006 | 0.017 | 0.024 | 0.023 | 0.000 | 0.000 | 0.020 | 0.000 | 0.017 | 0.027 | 0.048 | 0.025 |
| COCH | 0.007 | 0.052 | 0.026 | 0.029 | 0.029 | 0.048 | 0.031 | 0.033 | 0.020 | 0.011 | 0.000 | 0.000 |
| KISS | 0.021 | 0.030 | 0.033 | 0.026 | 0.012 | 0.019 | 0.049 | 0.033 | 0.007 | 0.009 | 0.016 | 0.012 |
| NARE | 0.030 | 0.041 | 0.024 | 0.028 | 0.036 | 0.051 | 0.039 | 0.045 | 0.023 | 0.012 | 0.000 | 0.012 |
| WHEA | 0.027 | 0.044 | 0.037 | 0.050 | 0.042 | 0.053 | 0.084 | 0.057 | 0.023 | 0.023 | 0.015 | 0.020 |
| WAWI | 0.033 | 0.034 | 0.027 | 0.018 | 0.025 | 0.027 | 0.036 | 0.035 | 0.012 | 0.012 | 0.001 | 0.009 |
| WABO | 0.027 | 0.026 | 0.022 | 0.007 | 0.019 | 0.017 | 0.033 | 0.030 | 0.005 | 0.012 | 0.005 | 0.010 |
| BOG | 0.091 | 0.092 | 0.089 | 0.056 | 0.057 | 0.038 | 0.092 | 0.076 | 0.060 | 0.072 | 0.069 | 0.068 |
| INTE | 0.100 | 0.092 | 0.092 | 0.059 | 0.058 | 0.041 | 0.087 | 0.074 | 0.066 | 0.073 | 0.073 | 0.069 |
| NORW | 0.007 | 0.033 | 0.011 | 0.011 | 0.020 | 0.058 | 0.006 | 0.028 | 0.013 | 0.005 | 0.000 | 0.000 |
| BERE | 0.011 | 0.037 | 0.011 | 0.027 | 0.036 | 0.084 | 0.009 | 0.048 | 0.028 | 0.032 | 0.011 | 0.028 |
| CHAR | 0.000 | 0.014 | 0.006 | 0.000 | 0.000 | 0.026 | 0.000 | 0.024 | 0.012 | 0.056 | 0.042 | 0.042 |
| ATIK | 0.009 | 0.007 | 0.019 | 0.057 | 0.007 | 0.030 | 0.063 | 0.010 | 0.039 | 0.034 | 0.058 | 0.068 |

1. Continued

|  | MOOS | KENO | KAPU | COCH | KISS | NARE | WHEA | WAWI | WABO | BOG | INTE | NORW |
| --- | --- | --- | --- | --- | --- | --- | --- | --- | --- | --- | --- | --- |
| WOOD | 0.021 | 0.028 | 0.030 | 0.039 | 0.045 | 0.036 | 0.027 | 0.022 | 0.034 | 0.043 | 0.060 | 0.026 |
| REDL | 0.035 | 0.040 | 0.027 | 0.042 | 0.070 | 0.047 | 0.033 | 0.027 | 0.035 | 0.044 | 0.063 | 0.058 |
| SIOU | 0.019 | 0.049 | 0.016 | 0.032 | 0.068 | 0.041 | 0.028 | 0.020 | 0.022 | 0.032 | 0.057 | 0.046 |
| IGNA | 0.037 | 0.041 | 0.035 | 0.041 | 0.051 | 0.034 | 0.027 | 0.024 | 0.030 | 0.050 | 0.064 | 0.037 |
| WABA | 0.016 | 0.032 | 0.017 | 0.034 | 0.034 | 0.021 | 0.022 | 0.027 | 0.030 | 0.031 | 0.040 | 0.039 |
| NIPI | 0.034 | 0.063 | 0.043 | 0.031 | 0.058 | 0.039 | 0.035 | 0.038 | 0.033 | 0.035 | 0.064 | 0.059 |
| KEEW | 0.007 | 0.036 | 0.014 | 0.019 | 0.041 | 0.041 | 0.032 | 0.020 | 0.021 | 0.028 | 0.060 | 0.017 |
| WEAG | 0.019 | 0.041 | 0.020 | 0.028 | 0.034 | 0.034 | 0.029 | 0.032 | 0.024 | 0.034 | 0.060 | 0.028 |
| BTL | 0.027 | 0.039 | 0.030 | 0.026 | 0.031 | 0.035 | 0.031 | 0.026 | 0.022 | 0.033 | 0.068 | 0.022 |
| WEBE | 0.009 | 0.035 | 0.002 | 0.016 | 0.029 | 0.021 | 0.018 | 0.013 | 0.022 | 0.041 | 0.064 | 0.014 |
| ATTA | 0.010 | 0.049 | 0.018 | 0.019 | 0.026 | 0.030 | 0.021 | 0.016 | 0.023 | 0.036 | 0.062 | 0.002 |
| MART | 0.012 | 0.026 | 0.000 | 0.014 | 0.035 | 0.029 | 0.024 | 0.014 | 0.022 | 0.039 | 0.065 | 0.014 |
| MOOS | 0.000 | 0.041 | 0.007 | 0.007 | 0.060 | 0.051 | 0.026 | 0.019 | 0.025 | 0.029 | 0.064 | 0.019 |
| KENO | 0.017 | 0.000 | 0.025 | 0.058 | 0.048 | 0.058 | 0.050 | 0.033 | 0.059 | 0.053 | 0.052 | 0.047 |
| KAPU | 0.000 | 0.001 | 0.000 | 0.012 | 0.055 | 0.045 | 0.037 | 0.019 | 0.033 | 0.034 | 0.056 | 0.031 |
| COCH | 0.007 | 0.061 | 0.030 | 0.000 | 0.052 | 0.043 | 0.032 | 0.027 | 0.027 | 0.032 | 0.066 | 0.033 |
| KISS | 0.008 | 0.063 | 0.020 | 0.036 | 0.000 | 0.021 | 0.035 | 0.040 | 0.047 | 0.057 | 0.062 | 0.043 |
| NARE | 0.032 | 0.064 | 0.046 | 0.026 | 0.030 | 0.000 | 0.012 | 0.023 | 0.032 | 0.055 | 0.066 | 0.039 |
| WHEA | 0.039 | 0.091 | 0.049 | 0.051 | 0.007 | 0.026 | 0.000 | 0.011 | 0.019 | 0.039 | 0.061 | 0.026 |
| WAWI | 0.020 | 0.051 | 0.027 | 0.028 | 0.013 | 0.009 | 0.016 | 0.000 | 0.012 | 0.028 | 0.053 | 0.021 |
| WABO | 0.019 | 0.049 | 0.024 | 0.032 | 0.010 | 0.015 | 0.012 | 0.000 | 0.000 | 0.033 | 0.074 | 0.024 |
| BOG | 0.058 | 0.075 | 0.058 | 0.080 | 0.057 | 0.077 | 0.073 | 0.029 | 0.031 | 0.000 | 0.028 | 0.057 |
| INTE | 0.061 | 0.066 | 0.055 | 0.087 | 0.064 | 0.089 | 0.081 | 0.039 | 0.042 | 0.000 | 0.000 | 0.095 |
| NORW | 0.009 | 0.051 | 0.033 | 0.013 | 0.034 | 0.003 | 0.046 | 0.013 | 0.016 | 0.085 | 0.084 | 0.000 |
| BERE | 0.033 | 0.089 | 0.067 | 0.040 | 0.076 | 0.026 | 0.093 | 0.036 | 0.039 | 0.117 | 0.115 | 0.000 |
| CHAR | 0.006 | 0.034 | 0.039 | 0.033 | 0.054 | 0.041 | 0.084 | 0.035 | 0.030 | 0.080 | 0.078 | 0.020 |
| ATIK | 0.039 | 0.043 | 0.033 | 0.080 | 0.035 | 0.054 | 0.041 | 0.045 | 0.039 | 0.088 | 0.086 | 0.071 |

1. Continued

|  | BERE | CHAR | ATIK |
| --- | --- | --- | --- |
| WOOD | 0.027 | 0.010 | 0.028 |
| REDL | 0.038 | 0.031 | 0.040 |
| SIOU | 0.016 | 0.016 | 0.029 |
| IGNA | 0.039 | 0.020 | 0.044 |
| WABA | 0.013 | 0.007 | 0.032 |
| NIPI | 0.028 | 0.034 | 0.038 |
| KEEW | 0.006 | 0.005 | 0.023 |
| WEAG | 0.005 | 0.019 | 0.023 |
| BTL | 0.017 | 0.027 | 0.025 |
| WEBE | 0.014 | 0.003 | 0.026 |
| ATTA | 0.018 | 0.011 | 0.010 |
| MART | 0.011 | 0.009 | 0.038 |
| MOOS | 0.021 | 0.009 | 0.024 |
| KENO | 0.054 | 0.036 | 0.057 |
| KAPU | 0.021 | 0.019 | 0.034 |
| COCH | 0.030 | 0.023 | 0.040 |
| KISS | 0.056 | 0.042 | 0.059 |
| NARE | 0.041 | 0.027 | 0.061 |
| WHEA | 0.039 | 0.019 | 0.039 |
| WAWI | 0.031 | 0.019 | 0.027 |
| WABO | 0.025 | 0.027 | 0.028 |
| BOG | 0.042 | 0.052 | 0.026 |
| INTE | 0.077 | 0.070 | 0.065 |
| NORW | 0.027 | 0.010 | 0.025 |
| BERE | 0.000 | 0.009 | 0.038 |
| CHAR | 0.022 | 0.000 | 0.037 |
| ATIK | 0.094 | 0.079 | 0.000 |

(b)

|  | TRC1 | TRC2 | TRC3 |
| --- | --- | --- | --- |
| TRC1 | 0.000 | 0.048 | 0.014 |
| TRC2 | 0.067 | 0.000 | 0.044 |
| TRC3 | 0.030 | 0.157 | 0.000 |

(c)

|  | TLC1 | TLC2 | TLC3 | TLC4 |
| --- | --- | --- | --- | --- |
| TLC1 | 0.000 | 0.016 | 0.016 | 0.041 |
| TLC2 | 0.017 | 0.000 | 0.028 | 0.055 |
| TLC3 | 0.028 | 0.024 | 0.000 | 0.045 |
| TLC4 | 0.138 | 0.096 | 0.071 | 0.000 |

**S1.5: Detailed description of network metric results**

The average clustering coefficient for the network weighted by R_ST_ was 0.73 and ranged from 0.00 for The Bog and North Interlake sampling locations to 1.00 for the Atiko and Kenogami sampling locations (Table S1.4). The average degree was 14.30 and ranged from one for the North Interlake sampling location to 24 for Big Trout Lake (Table S1.4). The average betweenness was 7.37 and ranged from 0.00 for the Atiko, Kenogami, and North Interlake sampling locations to 51.31 for the Wapisu-Wimapedi sampling location (Table S1.4). The mean of the average inverse edge weight across all nodes was 109.85 and ranged from 0.00 for the North Interlake sampling location to 370.89 for the Red Lake sampling location (Table S1.4).

The average clustering coefficient for the network weighted by F_ST_ was much lower (0.37) than for R_ST_, ranging from 0.00 for all disconnected components, as well as Atiko, Naosap-Reed, Nipigon, Red Lake, Wabowden, Wapisu-Wimapedi, and Wheadon, to 1.00 for the Big Trout Lake, Cochrane, Norway House, and Woodland Caribou Provincial Park sampling locations (Table S1.4). The average degree was 3.78 and ranged from 0.00 for all disconnected components to 11.00 for Webequie (Table S1.4). Average betweenness was 12.19 and ranged from 0.00 for all disconnected components, as well as Atiko, Big Trout Lake, Cochrane, Nipigon, Naosap-Reed, Norway House, Red Lake, Wabowden, and Woodland Caribou Provincial Park, to 84.38 for the Webequie sampling location (Table S1.4). The mean of the average inverse edge weight across all nodes was 140.20 and ranged from 0.00 for the Nipigon sampling locations to 325.88 for the Marten Falls sampling location (Table S1.4).

Table S1.4: Average inverse edge weight (AIEW), degree, clustering coefficient (CC), and betweenness centrality (BC) for networks with nodes based on the woodland caribou sampled areas in Ontario and Manitoba, Canada when edges are weighted with R_ST_ and F_ST_.

|  | R_ST_ | | | |  | F_ST_ | | | |
| --- | --- | --- | --- | --- | --- | --- | --- | --- | --- |
| Sampled  area | AIEW | Degree | CC | BC |  | AIEW | Degree | CC | BC |
| ATIK | 113.90 | 5 | 1.000 | 0.000 |  | 104.17 | 1 | 0.000 | 0.000 |
| ATTA | 105.37 | 17 | 0.743 | 5.030 |  | 313.65 | 10 | 0.444 | 32.067 |
| BERE | 57.09 | 10 | 0.844 | 0.797 |  | 146.21 | 4 | 0.833 | 0.167 |
| BOG | 17.18 | 2 | 0.000 | 25.000 |  |  | 0 | 0.000 | 0.000 |
| BTL | 211.21 | 22 | 0.684 | 10.984 |  | 178.01 | 5 | 1.000 | 0.000 |
| CHAR | 57.22 | 12 | 0.879 | 0.967 |  | 150.87 | 9 | 0.472 | 19.550 |
| COCH | 57.50 | 12 | 0.894 | 0.758 |  | 108.22 | 2 | 1.000 | 0.000 |
| IGNA | 176.43 | 18 | 0.693 | 7.172 |  |  | 0 | 0.000 | 0.000 |
| INTE | 0.00 | 1 | 0.000 | 0.000 |  |  | 0 | 0.000 | 0.000 |
| KAPU | 86.73 | 16 | 0.783 | 4.827 |  | 204.70 | 4 | 0.667 | 4.667 |
| KEEW | 151.42 | 15 | 0.829 | 1.875 |  | 140.05 | 10 | 0.533 | 32.950 |
| KENO | 189.95 | 6 | 1.000 | 0.000 |  |  | 0 | 0.000 | 0.000 |
| KISS | 86.88 | 13 | 0.833 | 1.550 |  |  | 0 | 0.000 | 0.000 |
| MART | 52.98 | 18 | 0.732 | 5.781 |  | 325.88 | 9 | 0.611 | 8.667 |
| MOOS | 145.24 | 20 | 0.721 | 8.811 |  | 120.10 | 7 | 0.619 | 16.500 |
| NARE | 74.37 | 12 | 0.803 | 1.851 |  | 84.03 | 1 | 0.000 | 0.000 |
| NIPI | 89.08 | 14 | 0.791 | 4.123 |  | 0.00 | 1 | 0.000 | 0.000 |
| NORW | 91.88 | 17 | 0.765 | 4.816 |  | 313.66 | 2 | 1.000 | 0.000 |
| REDL | 370.69 | 16 | 0.767 | 3.970 |  | 89.29 | 1 | 0.000 | 0.000 |
| SIOU | 122.68 | 21 | 0.681 | 13.251 |  | 85.33 | 3 | 0.333 | 20.000 |
| WABA | 88.42 | 21 | 0.671 | 14.078 |  | 72.83 | 6 | 0.400 | 29.133 |
| WABO | 81.04 | 17 | 0.779 | 3.779 |  | 84.75 | 1 | 0.000 | 0.000 |
| WAWI | 99.70 | 17 | 0.676 | 51.309 |  | 85.44 | 3 | 0.000 | 56.000 |
| WEAG | 91.11 | 15 | 0.752 | 4.533 |  | 135.02 | 7 | 0.667 | 4.917 |
| WEBE | 127.98 | 18 | 0.745 | 5.504 |  | 166.26 | 11 | 0.436 | 84.383 |
| WHEA | 63.30 | 9 | 0.917 | 0.437 |  | 89.64 | 2 | 0.000 | 20.000 |
| WOOD | 156.59 | 22 | 0.615 | 17.798 |  | 86.31 | 3 | 1.000 | 0.000 |

Table S1.5: Boreal caribou groupings delineated based on a) R_ST_ and b) F_ST_, using the multilevel (ML), leading eigenvector (LE), walktrap (WT), edge betweenness (EB), infomap (IM), label propogation (LP), and spinglass^*^ (SG) community detection algorithms. Colors indicate similarities in groupings among community detection algorithms.

(a)

| Sampled area | ML | LE | FG | WT | EB | IM | LP | SG |
| --- | --- | --- | --- | --- | --- | --- | --- | --- |
| ATTA | 1 | 2 | 1 | 2 | 1 | 1 | 1 | 3 |
| BTL | 1 | 2 | 1 | 2 | 1 | 1 | 1 | 3 |
| COCH | 1 | 2 | 1 | 2 | 1 | 1 | 1 | 3 |
| KISS | 1 | 2 | 1 | 2 | 1 | 1 | 1 | 3 |
| MART | 1 | 2 | 1 | 2 | 1 | 1 | 1 | 3 |
| NARE | 1 | 2 | 1 | 2 | 1 | 1 | 1 | 3 |
| WABO | 1 | 2 | 1 | 2 | 1 | 1 | 1 | 3 |
| WAWI | 1 | 2 | 1 | 3 | 1 | 1 | 1 | 3 |
| WEBE | 1 | 2 | 1 | 2 | 1 | 1 | 1 | 3 |
| WHEA | 1 | 2 | 1 | 2 | 1 | 1 | 1 | 3 |
| BOG | 2 | 2 | 1 | 4 | 2 | 2 | 2 | 1 |
| INTE | 2 | 2 | 1 | 5 | 2 | 2 | 2 | 1 |
| CHAR | 3 | 1 | 2 | 1 | 1 | 1 | 1 | 2 |
| IGNA | 3 | 1 | 2 | 2 | 1 | 1 | 1 | 2 |
| KEEW | 3 | 1 | 2 | 1 | 1 | 1 | 1 | 2 |
| SIOU | 3 | 1 | 2 | 2 | 1 | 1 | 1 | 2 |
| BERE | 3 | 2 | 1 | 2 | 1 | 1 | 1 | 3 |
| NORW | 3 | 2 | 1 | 2 | 1 | 1 | 1 | 3 |
| ATIK | 4 | 1 | 2 | 1 | 1 | 1 | 1 | 2 |
| KAPU | 4 | 1 | 2 | 1 | 1 | 1 | 1 | 2 |
| KENO | 4 | 1 | 2 | 1 | 1 | 1 | 1 | 2 |
| MOOS | 4 | 1 | 2 | 1 | 1 | 1 | 1 | 2 |
| NIPI | 4 | 1 | 2 | 1 | 1 | 1 | 1 | 2 |
| REDL | 4 | 1 | 2 | 1 | 1 | 1 | 1 | 2 |
| WABA | 4 | 1 | 2 | 1 | 1 | 1 | 1 | 2 |
| WEAG | 4 | 1 | 2 | 1 | 1 | 1 | 1 | 2 |
| WOOD | 4 | 1 | 2 | 1 | 1 | 1 | 1 | 2 |

(b)

| Sampled area | ML | LE | FG | WT | EB | IM | LP |
| --- | --- | --- | --- | --- | --- | --- | --- |
| BOG | 1 | 2 | 5 | 6 | 3 | 4 | 3 |
| NARE | 2 | 8 | 4 | 4 | 2 | 2 | 2 |
| WABO | 2 | 8 | 4 | 3 | 2 | 2 | 2 |
| WAWI | 2 | 8 | 4 | 3 | 2 | 2 | 2 |
| WHEA | 2 | 8 | 4 | 4 | 2 | 2 | 2 |
| COCH | 3 | 1 | 2 | 5 | 1 | 1 | 1 |
| KAPU | 3 | 1 | 2 | 5 | 1 | 1 | 1 |
| MOOS | 3 | 1 | 2 | 5 | 1 | 1 | 1 |
| WEBE | 3 | 8 | 2 | 1 | 1 | 1 | 1 |
| INTE | 4 | 3 | 6 | 7 | 4 | 5 | 4 |
| IGNA | 5 | 4 | 7 | 8 | 5 | 6 | 5 |
| NIPI | 6 | 7 | 1 | 10 | 1 | 1 | 1 |
| REDL | 6 | 7 | 1 | 2 | 6 | 3 | 1 |
| SIOU | 6 | 7 | 1 | 2 | 6 | 3 | 1 |
| WABA | 6 | 7 | 1 | 1 | 1 | 1 | 1 |
| BERE | 7 | 7 | 1 | 1 | 1 | 1 | 1 |
| BTL | 7 | 7 | 1 | 1 | 1 | 1 | 1 |
| KEEW | 7 | 7 | 1 | 1 | 1 | 1 | 1 |
| MART | 7 | 1 | 2 | 1 | 1 | 1 | 1 |
| WEAG | 7 | 7 | 1 | 1 | 1 | 1 | 1 |
| KISS | 8 | 5 | 8 | 9 | 7 | 7 | 6 |
| ATIK | 9 | 9 | 3 | 1 | 1 | 1 | 1 |
| ATTA | 9 | 9 | 3 | 1 | 1 | 1 | 1 |
| CHAR | 9 | 9 | 3 | 1 | 1 | 1 | 1 |
| NORW | 9 | 9 | 3 | 1 | 1 | 1 | 1 |
| WOOD | 9 | 9 | 3 | 1 | 1 | 1 | 1 |
| KENO | 10 | 6 | 9 | 11 | 8 | 8 | 7 |

^*^The spinglass community detection algorithm cannot delineate communities in networks with disconnected nodes and was, therefore, not computed for the F_ST_ network.

**References**

Blondel, V.D., Guillaume, J.L., Lambiotte, R., & Lefebvre, E. (2008) Fast unfolding of communities in large networks. *Journal of Statistical Mechanics: Theory and Experiment*, **2008**.

Clauset, A., Newman, M.E.J., & Moore, C. (2004) Finding community structure in very large networks. *Physical Review E - Statistical Physics, Plasmas, Fluids, and Related Interdisciplinary Topics*, **70**, 066111.

Cornuet, J.M. & Luikart, G. (1996) Description and power analysis of two tests for detecting recent population bottlenecks from allele frequency data. *Genetics*, **144**, 2001–2014.

Ecological Stratification Working Group (1995) A national ecological framework for Canada. Agriculture and Agri-Food Canada, Research Branch, Centre for Land and Biological Resources Research and Environment Canada, State of the Environment Directorate, Ecozone Analysis Branch, Ottawa/Hull.

Environment Canada (2012) Recovery Strategy for the Woodland Caribou (*Rangifer tarandus caribou*), Boreal population, in Canada. Species at Risk Act Recovery Strategy Series. Environment Canada, Ottawa. xi + 138pp.

Evanno, G., Regnaut, S., & Goudet, J. (2005) Detecting the number of clusters of individuals using the software STRUCTURE: A simulation study. *Molecular Ecology*, **14**, 2611–2620.

Freeman, L.C. (1977) A Set of Measures of Centrality Based on Betweenness. *Sociometry*, **40**, 35–41.

Garza, J.C. & Williamson, E.G. (2001) Detection of reduction in population size using data from microsatellite loci. *Molecular Ecology*, **10**, 305–318.

Girvan, M. & Newman, M.E.J. (2002) Community Structure in Social and Biological Networks. *Proceedings of the National Academy of Sciences of the United States of America*, **99**, 7821–7826.

Johnson, E.A. (1992) *Fire and Vegetation Dynamics: Studies from the North American Boreal Forest.* Cambridge University Press, Cambridge, UK.

Kivelä, M., Arnaud-Haond, S., & Saramäki, J. (2015) EDENetworks: A user-friendly software to build and analyse networks in biogeography, ecology and population genetics. *Molecular Ecology Resources*, **15**, 117–122.

Koen, E.L., Bowman, J., & Wilson, P.J. (2016) Node-based measures of connectivity in genetic networks. *Molecular Ecology Resources*, **16**, 69–79.

Newman, M.E.J. (2006) Finding community structure in networks using the eigenvectors of matrices. *Physical Review E - Statistical, Nonlinear, and Soft Matter Physics*, **74**, 036104.

Peery, Z.M., Kirby, R., Reid, B.N., Stoelting, R., Doucet-Bëer, E., Robinson, S., Vásquez-Carrillo, C., Pauli, J.N., & Palsboll, P.J. (2012) Reliability of genetic bottleneck tests for detecting recent population declines. *Molecular Ecology*, **21**, 3403–3418.

Piry, S., Luikart, G., & Cornuet, J.M. (1999) BOTTLENECK: A computer program for detecting recent reductions in the effective population size using allele frequency data. *Journal of Heredity*, **90**, 502–503.

Pons, P. & Latapy, M. (2005) Computing communities in large networks using random walks. *Lecture Notes in Computer Science* (ed. by P. Yolum, T. Güngör, F. Gürgen, and C. Özturan), pp. 284–293. Springer, Berlin, Heidelberg.

Raghavan, U.N., Albert, R., & Kumara, S. (2007) Near linear time algorithm to detect community structures in large-scale networks. *Physical Review E - Statistical, Nonlinear, and Soft Matter Physics*, **76**, 036106.

Reichardt, J. & Bornholdt, S. (2006) When are networks truly modular? *Physica D: Nonlinear Phenomena*, **224**, 20–26.

Rosvall, M., Axelsson, D., & Bergstrom, C.T. (2009) The map equation. *European Physical Journal: Special Topics*, **178**, 13–23.

Rosvall, M. & Bergstrom, C.T. (2007) Maps of random walks on complex networks reveal community structure. *Proceedings of the National Academy of Sciences of the United States of America*, **105**, 1118–1123.

Watts, D.J. & Strogatz, S.H. (1998) Collective dynamics of ‘small-world’ networks. *Nature*, **393**, 440–442.
